# Identification and Characterization of the MADS-Box Genes and Their Contribution to Flower Organ in Carnation (*Dianthus caryophyllus* L.)

**DOI:** 10.3390/genes9040193

**Published:** 2018-04-04

**Authors:** Xiaoni Zhang, Qijian Wang, Shaozong Yang, Shengnan Lin, Manzhu Bao, Mohammed Bendahmane, Quanshu Wu, Caiyun Wang, Xiaopeng Fu

**Affiliations:** 1 Key Laboratory of Horticultural Plant Biology, College of Horticulture and Forestry Sciences, Huazhong Agriculture University, Wuhan 430070, China; zhangxiaoni@webmail.hzau.edu.cn (X.Z.); qjwang@webmail.hzau.edu.cn (Q.W.); yangshaozong@webmail.hzau.edu.cn (S.Y.); linshengnan@webmail.hzau.edu.cn (S.L.); mzbao@mail.hzau.edu.cn (M.B.); QuanshuWu@webmail.hzau.edu.cn (Qu.W.); wangcy@mail.hzau.edu.cn (C.W.); 2 Key Laboratory of Urban Agriculture in Central China (pilot run), Ministry of Agriculture, Wuhan 430070, China; 3 Laboratoire Reproduction et Development des Plantes, INRA-CNRS-Lyon1-ENS, Ecole Normale Supérieure Lyon, Lyon 520074, France; mohammed.bendahmane@ens-lyon.fr

**Keywords:** floral organs identity, *Dianthus caryophyllus* L., MADS-box genes, ABC model

## Abstract

*Dianthus* is a large genus containing many species with high ornamental economic value. Extensive breeding strategies permitted an exploration of an improvement in the quality of cultivated carnation, particularly in flowers. However, little is known on the molecular mechanisms of flower development in carnation. Here, we report the identification and description of MADS-box genes in carnation (*DcaMADS*) with a focus on those involved in flower development and organ identity determination. In this study, 39 MADS-box genes were identified from the carnation genome and transcriptome by the phylogenetic analysis. These genes were categorized into four subgroups (30 MIKC^c^, two MIKC*, two Mα, and five Mγ). The MADS-box domain, gene structure, and conserved motif compositions of the carnation MADS genes were analysed. Meanwhile, the expression of *DcaMADS* genes were significantly different in stems, leaves, and flower buds. Further studies were carried out for exploring the expression of *DcaMADS* genes in individual flower organs, and some crucial *DcaMADS* genes correlated with their putative function were validated. Finally, a new expression pattern of *DcaMADS* genes in flower organs of carnation was provided: sepal (three class E genes and two class A genes), petal (two class B genes, two class E genes, and one SHORT VEGETATIVE PHASE (*SVP*)), stamen (two class B genes, two class E genes, and two class C), styles (two class E genes and two class C), and ovary (two class E genes, two class C, one AGAMOUS-LIKE 6 (*AGL6*), one SEEDSTICK (*STK*), one *B sister*, one *SVP*, and one *Mα*). This result proposes a model in floral organ identity of carnation and it may be helpful to further explore the molecular mechanism of flower organ identity in carnation.

## 1. Introduction

The MADS-box gene family playing an important role in the regulation of plant growth and development is well known as a key transcription factor (TF). MADS-box genes identified as floral homeotic genes contain a highly-conserved MADS box DNA-binding domain of approximately 58–60 amino-acid sequences in the N-terminal region, which bind to CArG boxes (CC[A/T]GG) [1,2,3,4]. MADS-box genes were classified into two major types: type I and type II genes, based on the phylogenetic relation of the conserved MADS box domain [5,6]. Sixty-two type I and 46 type II genes were identified and characterized in *Arabidopsis thaliana* [7]. Among them, type I genes can be further divided into three subgroups, Mα, Mβ, and Mγ, while type II, known as M-type, can be classified into two subgroups, MIKC^c^ and MIKC*, based on their structural characteristics [8,9]. It has been reported that type I MADS-box genes encode SRF-like domain proteins, that type II group genes encode MEF2-like genes of animals and yeast, and that MIKC-type genes are found only in plants [10,11]. The MIKC^c^ proteins contain four common domains, including MADS (M), weakly-conserved intervening (I), conserved keratin (K), and the highly-variable C-terminal (C) domain, which usually contains conserved subfamily-specific sequence motifs [12]. I domain is responsible for DNA binding specificity and dimerization of these proteins. In addition, K domain contributes to mediating dimerization, and C domain functions in transcriptional activation and in the formation of multimeric protein complexes. Compared with type II, type I group genes show a relatively simple gene structure. They are shorter, and usually only have one or two exons without the K domain [13]. With further study of MIKC^c^ type genes, they were subclassified into 12 groups, based on their phylogenetic relationships in *A. thaliana*. Nevertheless, the knowledge of the function of type I genes remains limited.

In plants, previous reports reveal that the MADS-box family plays a vital role in many developmental processes, such as flower organ identity [14], control of flowering time [15,16,17,18,19], fruit ripening [20], and the development of vegetative organs [21]. Moreover, MIKC^C^ -type MADS-box genes are involved in flowering time control and floral organ identity. The well-known ABC model of flowers, which explain different floral organs identities, are controlled by the combinations of various types of genes [14]. Subsequently, ABC model developed into ABCDE model: sepals (A + E), petals (A + B + E), stamens (B + C + E), carpels (C + E), and ovules (D + E) [22]. In the newly-developed model, class A contains APETALA1 and FRUITFULL (*AP1* and *FUL*); class B contains PISTILLATA and APETALA3 (*PI* and *AP3*); class C contains AGAMOUS (*AG*); class D contains *STK*; and class E contains SEPALLATA genes 1–4 (*SEP1*, *SEP2*, *SEP3*, and *SEP4*) [22].

In addition to class ABCDE genes, there are many other MADS genes in regulating flower development in *A. thaliana*, such as, FLOWERING LOCUS C (*FLC*), SUPPRESSOR OF OVEREXPRESSION OF CO1 (*SOC1*), *SVP*, AGAMOUS-LIKE 24 (*AGL24*) [17], MADS AFFECTING FLOWERING (*MAF1/FLM*) [23] and AGAMOUS-LIKE 15/18 (*AGL15/AGL18*) [19]. Among them, the *FLC* gene encoding a specific MADS domain protein has the function of inhibiting flowering [24]. The *SOC1* gene can also regulate the flowering time by acting on the vernalization pathway [15]. *SVP* is considered as an important control factor of flowering time influenced by ambient temperature [16]. Moreover, the *AGL16* gene targeted by microRNA 824 (miR824) contributes to the repression of plant flowering time [18]. *AGL17* genes show unusually diverse expression patterns with member genes expressed in roots, in pollen [25], and in both [26]. These genes function as either positive (*SOC1*, *AGL24*) or negative regulators (*FLC*, *SVP*) of flower meristem identity genes together with other subfamilies, such as *AGL15*, *AGL12*, and *AGL17*. By contrast, type I genes were reported to only participate in the development of seed and female gametophyte [27,28,29].

The carnation is one of the most popular flowers [30]. If more flower shapes can be developed and the flowering time and flower morphology is easier to control, the economic value of carnation will be greatly enhanced. Thus, it is extremely essential to explore how the MADS-box gene family controls floral organ development and regulates flowering time. Recently, more and more MADS-box genes were identified and characterized in various plant species, such as *Arabidopsis* [7], tomato [31], rice [32], maize [33], cucumber [34], soybean [35], Chinese cabbage [36], sesame [37], and radish [38]. However, few studies of the genome-wide characterization of MADS-box genes in carnation were available. Fortunately, the advent of the carnation genome sequencing makes it possible to analyse MADS-box genes [39]. In this study, MADS-box members from carnation genome were systematically analysed and their gene structures, conserved motifs, phylogeny, and subcellular localization were presented for the first time. Additionally, preliminary prediction of gene functions were also verified, and the expression of the MADS-box family in carnation were detected with real-time PCR (RT-PCR). These results will offer an insight into the molecular mechanisms underlying flowering and floral organogenesis in carnation through analysis of the expression pattern of MADS-box genes.

## 2. Material and Methods

### 2.1. The Identification of MADS-Box Genes in Carnation’s Genome

We download 101 MADS-Box family genes in *Arabidopsis* from the TAIR website [40] in Table S1 and 71 MADS-Box family genes in the rice genome from the Rice Genome Annotation Project [41], respectively, in Table S2. All the downloaded protein sequences of MADS-Box family genes were used as query sequences by blastp searches (*e*-value 1^e-6^) against the carnation’s protein sequences to predict the carnation MADS-Box family genes. In addition, the Hidden Markov Model (HMM) profile for the MADS-box domain (Pfam accession number: PF00319) was also used to search (*e*-value 1^e-7^, score 30) against the genome protein sequences by using HMM search tool to ensure the completeness of MADS-box genes as far as possible. Then the genes obtained by the two methods mentioned above ere intersected to guarantee the appropriate selection of genes. Each gene predicted was subsequently verified through the National Center for Biotechnology Information (NCBI) [42], SMART [43] and Pfam database [44] to confirm the completeness of the MADS-box domain.

### 2.2. Conserved Sequence and Structure Model Analysis

To search for the conserved motifs localized within the 39 DcaMADS protein sequences, the multiple expectation for motif elicitation (MEME) tool [45] was used with default parameters. A maximum of 15 motifs were searched. This study took advantage of carnation genome annotation file [46] to extract the carnation MADS-box gene structure information, and then the picture was drawn with the R language.

### 2.3. Phylogenetic Analysis of MADS-Box Proteins

Multiple sequence alignments were performed between MADS-box protein sequences from carnation, *Arabidopsis*, and rice by using the ClustalX-2.0 software package with default parameters [47]. A phylogenetic tree was constructed with aligned MADS-box protein sequences with Clustal X by using the neighbour-joining (NJ) method with 1000 iterations for the bootstrap values. The rectangular phylogenetic tree was generated using MEGA6 software package [48].

### 2.4. RNA Sample Preparation and Quantitative PCR Expression Analysis

Young stems, young leaves, flower buds (diameter 0.5–0.8 cm), and individual flower organs (before blooming) of the carnation cultivar *Master* were collected for gene expression assays with quantitative real-time RT-PCR (qRT-PCR). The carnation cultivar *Master* was planted in the experiment yard of Huazhong Agriculture University (Wuhan, China). Total RNA of each sample was extracted using an EASYspin Plant RNA kit reagent (Aidlab Biotechnologies, Beijing, China) according to the manufacturer’s instructions. The PCR amplification was carried out in a 96-well plate with the following cycling parameters: heating for 2 min at 95 °C, 40 cycles of denaturation at 95 °C for 10 s, annealing for 20 s at 60 °C, and extension at 72 °C for 35 s. Three biological replicates were included per sample. The qRT-PCR was conducted using SYBR Primix Ex Taq kit (TaKaRa, Dalian, China) in an Applied Biosystems Real-Time PCR System (Life Technologies, Carlsbad, CA, USA). To confirm results’ reliability, each sample was conducted with three biological and three technical replicates. The housekeeping gene *DcaGAPDH* (glyceraldehyde-3-phosphate dehydrogenase) was selected as an internal quantitative control (Table S3). The relative expression values were calculated using the comparative CT(2^−^^△△^^CT^) method. The primers used in the analysis are listed in Table S3.

### 2.5. Subcellular Localization

The full-length candidate complementary DNA (cDNA) sequence was amplified from cDNA of the carnation cultivar *Master* by PCR. A suitable restriction site sequence was added to the ends of the primers. The primers used in the analysis were listed in Table S4. Products were cloned into vector plGFP 1301 using dual-enzyme digestion. Plasmids were isolated and transformed into *Nicotiana benthamiana* by injection transformation [49]. The transformed leaves were incubated for three days, then observed and photographed on a microscope (BX61, Olympus, Tokyo, Japan).

## 3. Results 

### 3.1. The Identification and Annotation Information of Carnation MADS-Box Domain Genes 

To define the candidate MADS-box genes, carnation genome and transcriptome protein sequences were searched by using a HMM profile in the Pfam database. A total of 46 putative genes in carnation were identified and all these candidate carnation MADS-box proteins were named DcaMADS1 to DcaMADS46 (Table 1), respectively. To ensure the reliability of these sequences, all the identified sequences were verified through the public databases, including NCBI, Pfam, and SMART, and searching these protein sequences against *A. thaliana* on the TAIR database by BLASTP. Among them, the complete open reading frames (ORF) sequences of five genes (*DcaMADS6*, *DcaMADS8*, *DcaMADS18*, *DcaMADS28*, and *DcaMADS29*) were obtained in the carnation transcriptome (Figure S1). In this study, the *DcaMADS8* and *DcaMADS31* genes’ names were still used for uniformity, while they were named, respectively, *CMB1* and *CMB2* [50]. Although *DcaMADS36* and *DcaMADS39* share the SRF-like domain, their sequence structure does not have a specific motif. Therefore, they are not suitable to be classified into any subgroup of MADS-box gene family, and they were eliminated from further analysis. Interestingly, three candidate genes. *DcaMADS3*, *DcaMADS4*, and *DcaMADS5*, display no difference in their protein sequence; consequently, only *DcaMADS3* sequence was selected for further study. A total of 39 sequences were selected in carnation without sequences incompletion. Compared with other species, carnation had a relatively small number of MADS-box gene families; 39 MADS-box genes were categorized into four subgroups (30 MIKC^c^, 2 MIKC*, 2 Mα, 5 Mγ) and 30 MIKC^C^-type proteins were subdivided into 12 subclasses.

### 3.2. Analysis of the Gene Structure and Conserved Sequence 

**Figure 1:**
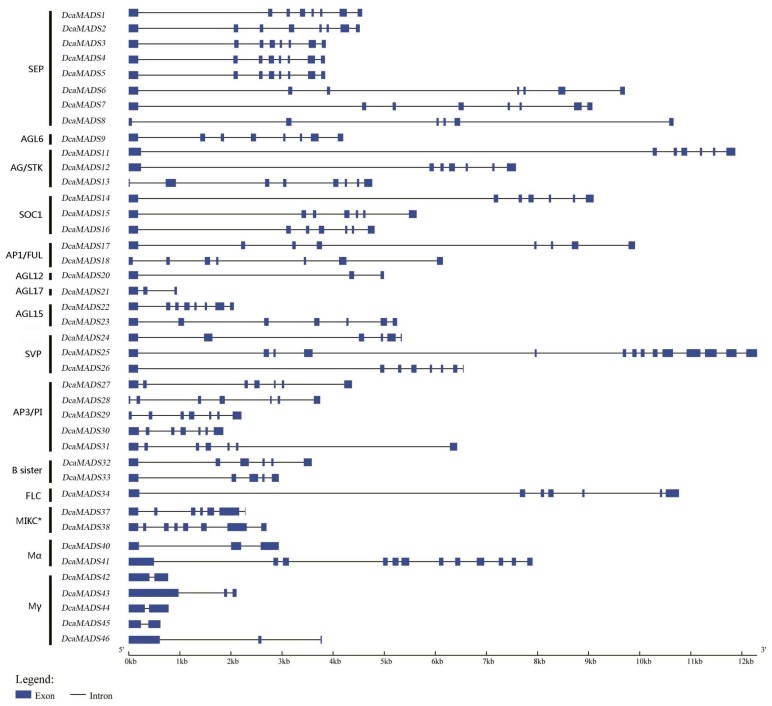
The exon-intron structure of *DcaMADS* genes. The lines indicate introns, and the blue boxes indicate exons.

**Figure 2:**
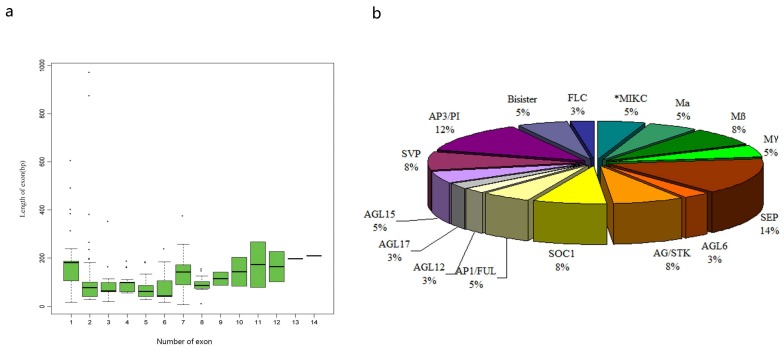
(**a**) Exon length distribution analysis of the carnation MADS-box genes. Exon length values were extracted from the carnation genome annotation file and then drawn the boxplot with the R language. Each box represents the exon size range in which 50% of the values for particular exon are grouped. The median is shown as a black line. (**b**) The classification and proportions of *DcaMADS* genes.

To better understand the structural diversity and gene evolutionary relationship, the intron–exon pattern of coding sequences of individual MADS-box genes in carnation was analysed (Figure 1). Many previous studies reported that type II genes contained multiple introns, whereas *Mα*, *Mβ*, and *Mγ* genes usually contained fewer of them [32,51]. This study obtained the similar findings that *Mα* and *Mγ* genes contained small numbers of introns in carnation, with the exception of *DcaMADS41*, containing as many as 11 introns. The structure of type II gene was more complex than that of type I in carnation. The maximum of 13 introns was observed in a single gene in *DcaMADS25* (SVP). The structure of typical MIKC^C^ genes with 1–6 exons and conserved C-terminal motifs in carnation were found, which was similar with previous study [52]. To further understand the intron–exon structure of carnation MIKC^C^ genes, we analysed the length of MIKC^C^ genes (Figure 2a). The result indicated that lengths of exon 3, 4, 5, and 8 between genes were consistent and stable, whereas the lengths of exon 1, 2, 6, and 7 were not. The presence of exons 9 or 10, 11, 12, 13, and 14 were peculiar to some specific genes.

**Figure 3:**
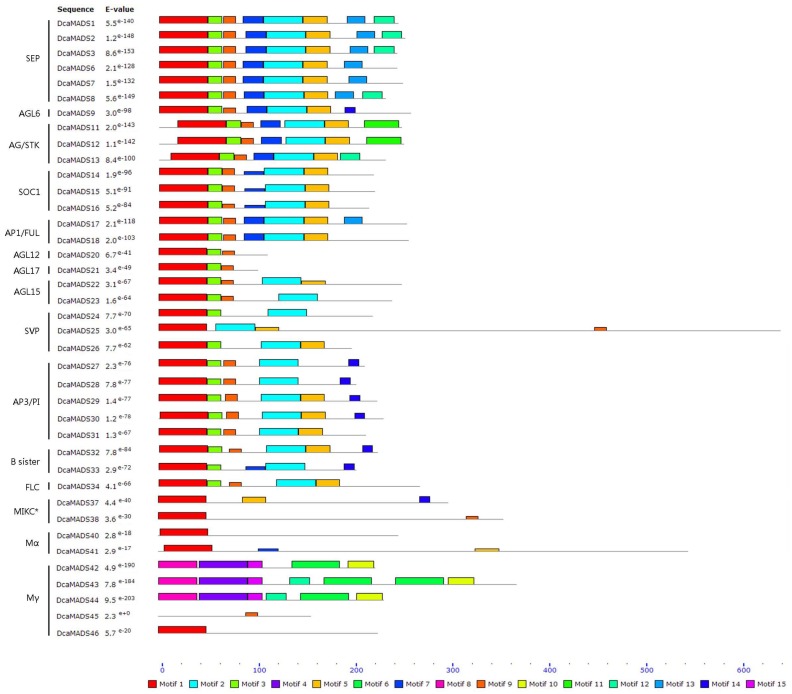
Conserved motifs of 39 carnation MADS-box proteins identified using the multiple expectation for motif elicitation (MEME) program. Motifs 1 to 15 are indicated by different coloured boxes. The names of all members and combined probability values are shown on the left side; motif sizes are shown at the bottom.

All 39 DcaMADS proteins were identify the motifs by the MEME motif search tool [53]. In total, 15 motifs were identified and were named motif 1–15 (Figure 3 and Figure S2). Among these motifs, the conserved motifs 1 and 3, which specify the MADS domain, were observed in most DcaMADS proteins. This motif KR[K/R]X4KK (motif 1 (Figure S2)) at positions 22–30 of the MADS-box domain plays an important role in the translocation of MADS-box proteins into the nucleus [54]. Motif 2 and 5, which specifies the K domain, were found in most MIKC^C^ group proteins. The sequences of DcaMADS20, DcaMADS21, DcaMADS42, DcaMADS43, and DcaMADS44 and DcaMADS45 proteins were incomplete, with DcaMADS20 and DcaMADS21 lacking K domain and DcaMADS42, 43, 44 and 45 lacking the MADS domain. Interestingly, only DcaMADS42, DcaMADS43, and DcaMADS44 proteins shared the same 4, 6, 8 and 10 motifs (Figure 3).

### 3.3. Phylogenetic Analysis of Carnation MADS-Box Domain

**Figure 4:**
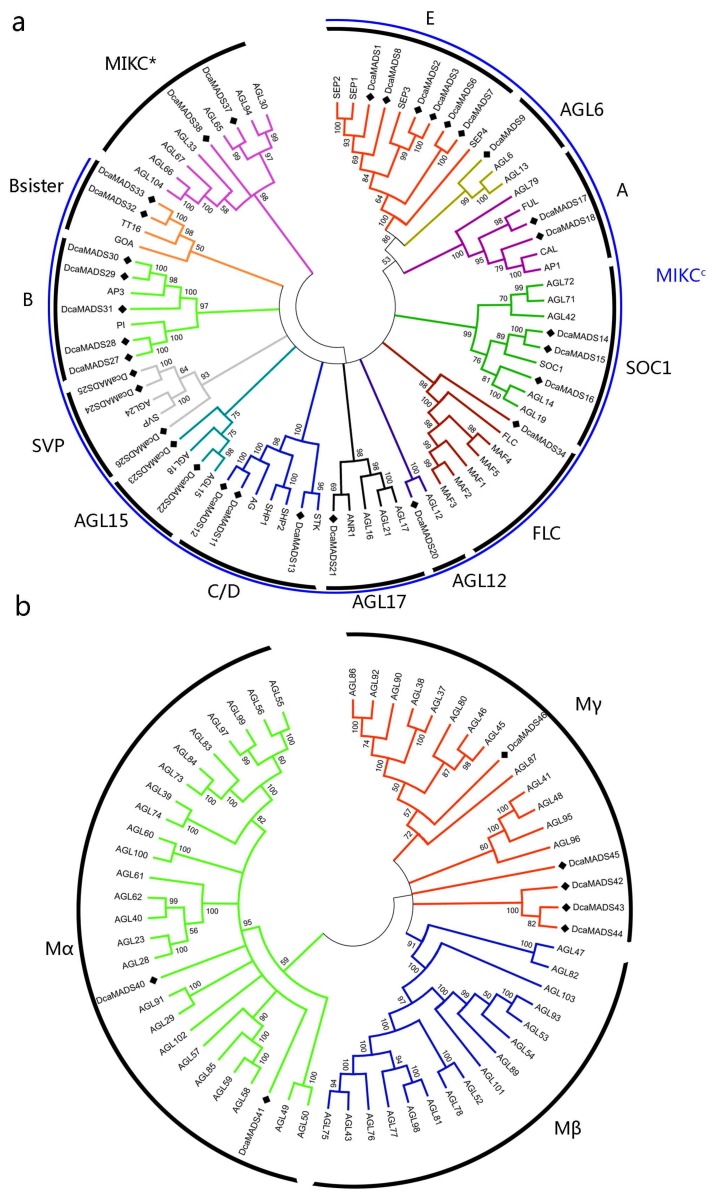
The phylogenetic tree of the 39 *DcaMADS* proteins was generated by the neighbour-joining (NJ) algorithm with 1000 iterations for the bootstrap values using Molecular Evolutionary Genetics Analysis (MEGA version 6.0) software [48]. The subgroups are marked in different colours. (**a**) Phylogenetic tree of *Dianthus caryophyllus* and *Arabidopsis* type II proteins. (**b**) Phylogenetic tree of *Arabidopsis* and *D. caryophyllus* type I proteins.

To know more about the phylogenetic relationships among carnation MADS-box genes, a phylogenetic tree between 39 carnation MADS-box genes and 101 *A. thaliana* MADS-box genes was constructed by NJ method (Table S1). It is obvious that *DcaMADS* genes were divided into four clades with reference to the classification of the *A. thaliana*, and then they were named subfamilies MIKC^C^, MIKC*, Mα, and Mγ (Figure 4). Additionally, to further confirm phylogenetic relationships, another phylogenetic tree was constructed by using MADS-box proteins from carnation and rice (Figure S3 and Table S2). The result of two phylogenetic trees were same. Of the 39 DcaMADS proteins, 32 members from DcaMADS1 to DcaMADS38 with high similarity could be unambiguously classified into MIKC type II, whereas the remaining seven members (DcaMADS39-DcaMADS46) were classified into type I according to their relation with AGL proteins [7,32]. In type II, two proteins (DcaMADS37 and DcaMADS38) were subgrouped into MIKC^*^, whereas the other 30 members (DcaMADS1-DcaMADS35) were classified into MIKC^C^. These 30 MIKC^C^-type proteins were subdivied into 12 subclasses with E (SEP) having six members, AGL6 (one member), C/D (AG/STK) (three members), SOC1 (three members), A (AP1/FUL) (two members), AGL12 (one member), AGL17 (one member), AGL15 (two members), SVP (three members), and B (AP3/PI) (five members), B sister (two members) and FLC (one member). Type I proteins were further classified into two subgroups: Mα (two members) and Mγ (five members) (Figure 2b). The number of MADS genes from subgroup SEP and AP3/PI in carnation was larger than that in other species, like in tomato [31], in cucumber [34], and in petunia [55] (Table S5).

### 3.4. Expression of DcaMADS Genes in Floral Organs

**Figure 5:**
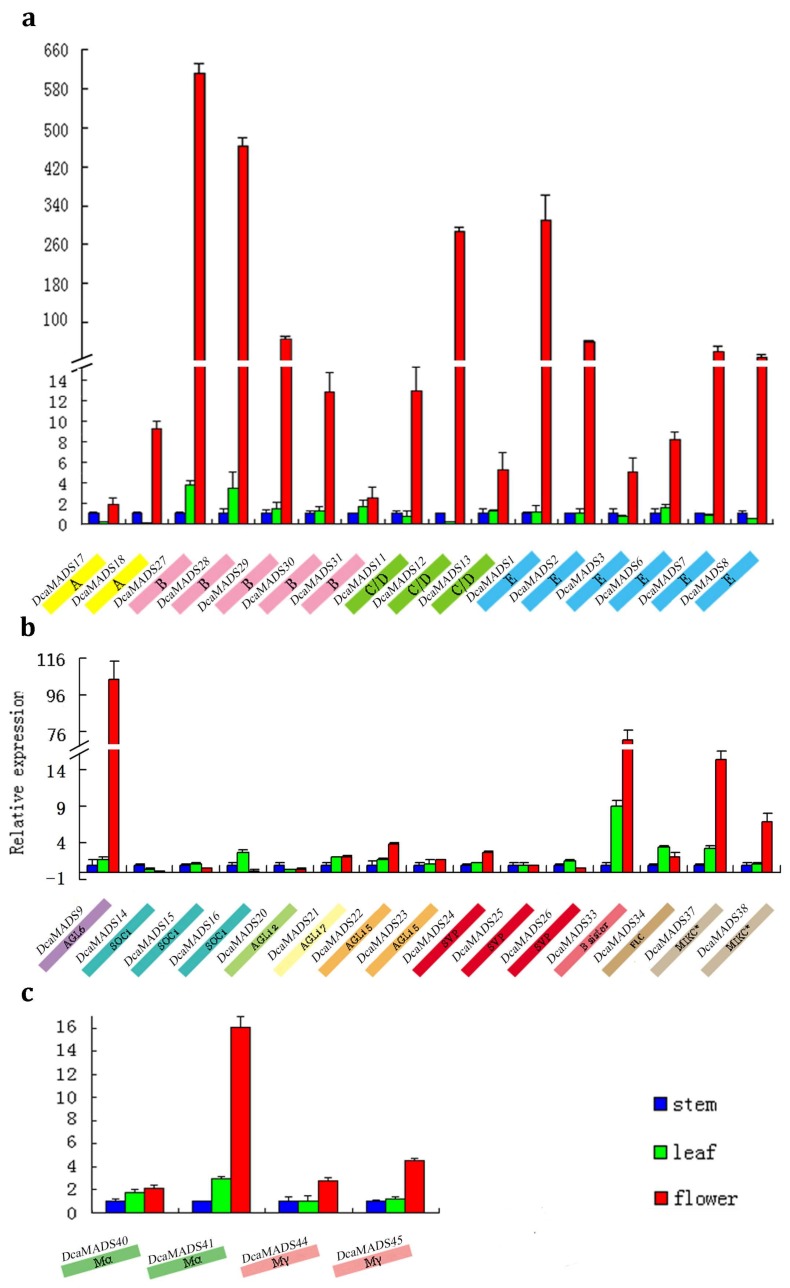
Expression analysis of 35 *DcaMADS* genes in different tissues, including stems, leaves, and flower buds. Each bar shows the standard deviation of triplicate assay. (**a**) The expression levels of 16 ABCDE *DcaMADS* genes which mean the class A, class B, class C/D and class E in the well-known ABC model of flowers. (**b**) The expression levels of genes in AGL6 (1), SOC1 (3), AGL12 (1), AGL17 (1), AGL15 (2), SVP (3), B sister (1), FLC (1), and MIKC^*^ (2) subgroups. (**c**) The expression levels of four type I *DcaMADS* genes.

MADS-box genes participate in various processes of plant growth and development. To know more about their expression patterns, the expression of 35 carnation MADS genes in stems, leaves, and flower buds were examined. No expression of *DcaMADS32*, *DcaMADS42*, *DcaMADS43*, and *DcaMADS46* genes in the above-mentioned samples was observed, which may be attributed to the fact that their expressions were too low to be detected. The expression level of A-, B-, C-, D-, and E class genes were higher in flower buds than that in stems and leaves (Figure 5), especially the genes *DcaMADS27*, *DcaMADS28*, *DcaMADS12*, and *DcaMADS1*. This was same with *DcaMADS9* (AGL6), *DcaMADS33* (B sister), and *DcaMADS37* (MIKC* groups). While only one gene in the Mα group, namely *DcaMADS41*, was highly expressed in flower buds. To obtain more knowledge the gene expression in five tissues involved in the development of reproductive organs were examined.

#### 3.4.1. SEPALLATA

**Figure 6:**
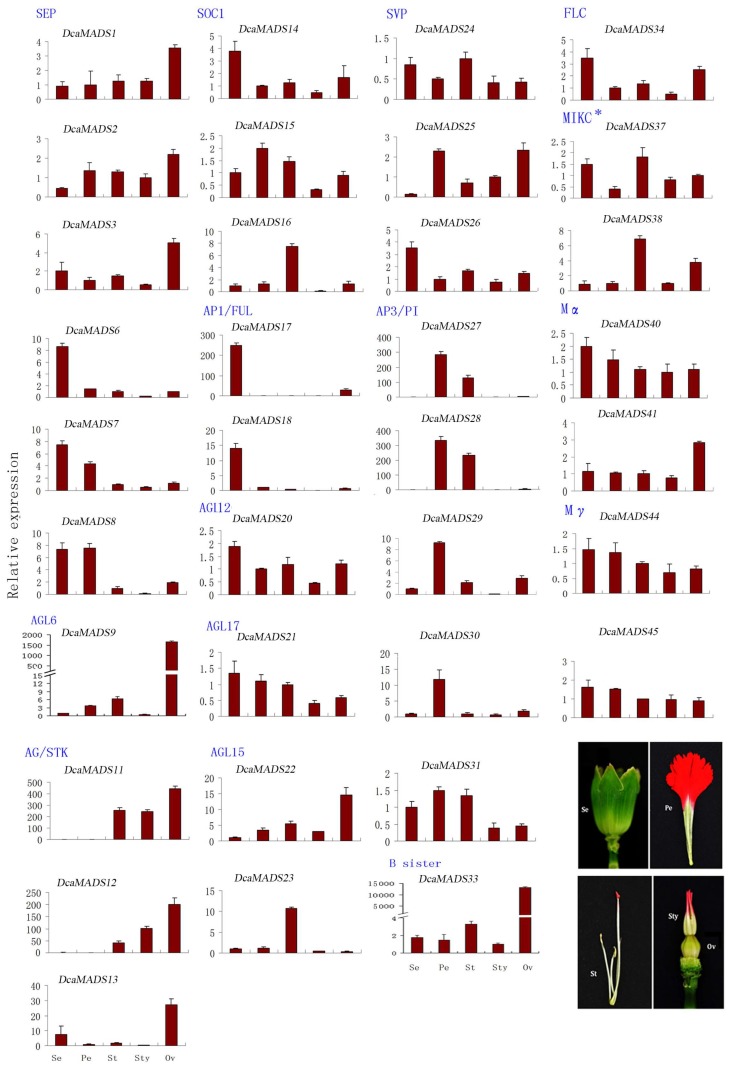
Organ specific expression analysis of 35 *DcaMADS* genes at different flower whorls. Se: sepals, Pe: petals, St: stamens, Sty: styles, Ov: ovaries. Each bar shows the standard deviation of triplicate assay.

E genes in the ABCE model play a significant role in floral organ development [55,56,57,58]. Six SEP (*DcaMADS1*, *DcaMADS2*, *DcaMADS3*, *DcaMADS6*, *DcaMADS7*, and *DcaMADS8*) genes were identified and analysed from carnation indicating that *DcaMADS1*, *DcaMADS2*, and *DcaMADS3* were highly expressed in ovary tissues. *DcaMADS6*, *DcaMADS7*, and *DcaMADS8* were expressed mainly in the sepals, and *DcaMADS7* and *DcaMADS8* were additionally expressed in petals (Figure 6).

#### 3.4.2. AGAMOUS-LIKE 6

There have been reports that AGL6 regulates floral organ identity [59]. We isolated one AGL6 gene (*DcaMADS9*) from carnation. The *DcaMADS9* gene had the highest expression in ovary tissues among five above-mentioned tissues. This finding was consistent to that of experiments with melon [60] (Figure 6).

#### 3.4.3. AGAMOUS/SEEDSTICK

The AG gene mainly functions in specifying stamen and carpel identity and STK in ovaries [61,62,63]. We identified two AG genes (*DcaMADS11* and *DcaMADS12*) that were expressed exclusively in stamens, styles, and ovaries, and the STK gene (*DcaMADS13*) exclusively in ovaries (Figure 6). These results are consistent with those found in other species plants, such as *Arabidopsis* [63], rice [64], tomato [61], etc., indicating that AG subfamily members specified stamen and carpel identity [7,31,32].

#### 3.4.4. SUPPRESSOR OF OVEREXPRESSION OF CO1

SOC1 is an important transcriptional regulation factor controlling flowering time [64,65]. We identified three SOC1 genes (*DcaMADS14*, *DcaMADS15*, and *DcaMADS16*) with various expression patterns in vegetative and reproductive organs of carnation. The *DcaMADS14* gene was found to be expressed primarily in sepals. The *DcaMADS15* gene was expressed in all tissues, but slightly higher in stamens and petals than in other tissues. Moreover, the *DcaMADS16* gene was only markedly detected in stamen tissues (Figure 6).

#### 3.4.5. APETALA 1/FRUITFUL

AP1/FUL genes are typical class A floral organ identity genes, which were expressed in inflorescence [66,67]. In addition, they are involved in specifying sepals and petals [68,69]. Two class A genes were identified (*DcaMADS17* and *DcaMADS18*) to have the same transcript patterns in stems and in leaves. However, they were highly expressed in sepals of carnation (Figure 6). Our results are also in line with the findings of one previous study reporting that the *FUL* gene was expressed in stems and leaves of *Arabidopsis* [70].

#### 3.4.6. AGAMOUS-LIKE 12

Only one *AGL12* gene (*DcaMADS20*) was detected in carnation. This gene was expressed in all tested organ tissues (Figure 5 and Figure 6), similar to their *Arabidopsis* counterpart AGL12 [21]. Moreover, AGL12 was also been detected to be strongly expressed in stems.

#### 3.4.7. AGAMOUS-LIKE17

We identified one AGL17 (*DcaMADS21*) gene which was reported with strong expression in different tissues, such as in roots [71], in pollen [25,72], or in leaf guard cells [68], and trichomes [5]. However, its expression level in all tissues of carnation merely displayed slight differences with low expression found in style and ovary tissues (Figure 6).

#### 3.4.8. AGAMOUS-LIKE 15

AGL15 can effectively regulate plants senescence in *Arabidopsis* [19]. In our study, two AGL15 genes (*DcaMADS22* and *DcaMADS23*) were detected and their expressions in different tissues were different. The gene of *DcaMADS22* was expressed mainly in ovary tissues, while *DcaMADS23* was specifically expressed in stamen tissues (Figure 6).

#### 3.4.9. SHORT VEGETATIVE PHASE/AGAMOUS-LIKE 24

Some genes from the same subfamily have different functions. For example, AGL24 (SVP subfamily) as a flowering promoter in Arabidopsis [16,17], while the SVP [72] gene in barley serves as a floral repressor. Three genes (*DcaMADS24, DcaMADS25*, and *DcaMADS26*) in this subfamily were found and their expressions in various organs were analysed (Figure 6). The two genes *DcaMADS24* and *DcaMADS26* were widely expressed in various tissues. *DcaMADS24* had a high expression level in stamens, while *DcaMADS26* high expressed in sepals. In addition, *DcaMADS25* was expressed in petal and ovary tissues.

#### 3.4.10. APETALA 3/PISTILLATA

B class genes play an important role in controlling petals and stamens during flower development [73,74]. We found two *PI* genes (*DcaMADS27* and *DcaMADS28*) and two *AP3* genes (*DcaMADS29* and *DcaMADS30*) that were expressed exclusively in the flower buds. *DcaMADS31* in carnation has a high homology with TM6 in tomato. Transcripts for these genes were abundant in the petals and stamens of carnation. We also found that relatively high expression of *DcaMADS27* and *DcaMADS28* and low expression of *DcaMADS29* and *DcaMADS30* in sepals and pistils (Figure 6). Moreover, *DcaMADS31* was expressed in all tissues, but it was relatively highly expressed in petals and stamens, compared with other tissues.

#### 3.4.11. B sister

*The DcaMADS33* gene was identified as a sister group of B genes and was, therefore, named B sister (Bs) genes [75,76]. GOA/TT16 is an *Arabidopsis* B sister gene and was reported to function in the endothelial cells and the seed coat controlling flavonoid biosynthesis [77,78]. *DcaMADS33* was found to be expressed strongly in ovary tissues in carnation, similar to the counterpart in *Arabidopsis* (Figure 6).

#### 3.4.12. FLOWERING LOCUS

The function of *FLC* gene is to inhibit flowering [79,80]. Five *FLC* genes were identified in *Arabidopsis* and in poplar [81], respectively. However, only one *FLC* gene (*DcaMADS34*) was detected in carnation, which was expressed in sepals of carnation (Figure 6).

#### 3.4.13. MIKC* 

Two MIKC* genes (*DcaMADS37* and *DcaMADS38*) were detected in genome of carnation. Both of them were found to be expressed in stamens, which are consistent with the findings in radish and *Arabidopsis* [38,82,83]. However, these genes showed differential expression in other tissues. For example, *DcaMADS37* was highly expressed in sepals, but *DcaMADS38* was not expressed in sepals. The two genes were expressed widely in other floral organ tissues (Figure 6).

#### 3.4.14. Mα

Two Mα genes (*DcaMADS40* and *DcaMADS41*) were detected in all flowers’ organs in this study. However, they exhibited distinct expression patterns. *DcaMADS40* had slightly higher expression in sepals than in other tissues, whereas *DcaMADS41* was found to have high expression only in ovaries (Figure 6).

#### 3.4.15. Mγ

This study isolated five Mγ genes (*DcaMADS42, DcaMADS43, DcaMADS44*, *DcaMADS45*, and *DcaMADS46*) through phylogenetic tree analysis. The two Mγ genes (*DcaMADS44* and *DcaMADS45*) were expressed in all of the flowers’ organs (Figure 6). Until now, there have been few reports on the function of the Mγ gene in plants.

### 3.5. Subcellular Localization of DcaMADS 

**Figure 7:**
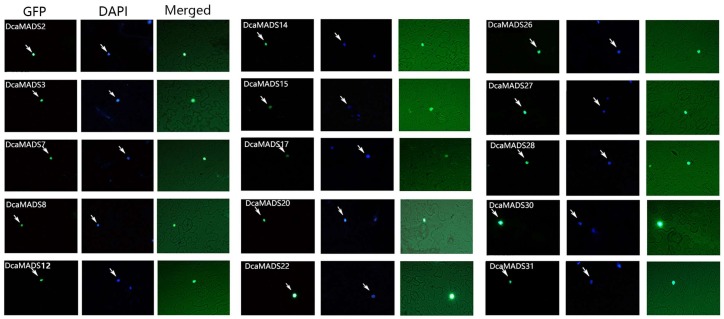
Subcellular localization of DcaMADS fused with green fluorescent protein. Plasmids containing fusions of GFP and DcaMADS driven by the CaMV35S promoter were transiently expressed.

The subcellular localization of DcaMADS proteins was investigated via injection transformation with green fluorescent protein (GFP) fused with 15 DcaMADS proteins in *N. benthamiana*, belonging to eight subfamilies (A, B, C, E, SOC1, AGL12, AGL15, and SVP subfamilies), which were highly expressed in flower organs. All of the GFP-DcaMADS signals were localized to nuclei (Figure 7). This result is similar to that found in the experiments with other plants, in which several MADS-boxes as transcription factors have been found to be localized to the nucleus [12,27,83,84]. The motif KR[K/R]X4KK (motif 1 (Figure S2)) at positions 22–30 of the MADS-box domain plays an important role in the translocation of MADS-box proteins into the nucleus [54]. This motif of DcaMADS proteins in carnation is highly conservative.

## 4. Discussion 

In recent years, more and more studies of the MADS-box family in various species, such as in *Arabidopsis* [7], poplar [81], rice [32], grape [85], cucumber [34], soybean [35], *Prunus mume* [86], apple [51], *Erycina pusilla* [87], *Brassica rapa* [36], and radish [38], have been reported. MADS-box genes in various species showed great difference. However, the MADS-box gene family in Caryophyllaceae has not been reported. In our study, seven Type I MADS-box genes (2 Mα, 5 Mγ) and 32 type II MADS-box genes (2 MIKC* and 30 MIKC^c^) were identified in carnation. The phylogenetic relationships and expression patterns of the two type genes varied greatly. This study will be hopeful to understanding *DcaMADS* genes’ contributions to organ development in carnation.

### 4.1. AP3/PI and SEP Subfamily with Duplication Influences Evolution and Divergence

AP3/PI subfamily: B function gene is one of the most frequently studied MADS-box gene controlling floral organ. The evolution of this subfamily gene involves a large number of gene duplication events at different taxonomic levels [88,89]. One large-scale gene duplication event occurs before the formation of angiosperms, resulting in two evolution lines of *paleoAP3* and *PI*. The other gene duplication event occurs before the formation of core eudicot plants, giving rise to the birth of two evolution lines of *euAP3* and *TM6* deriving from the *paleoAP3* gene through replication [90,91]. These different evolution lines can be distinguished by their C-terminal domain motifs. The C-terminal domain of *TM6* gene is found to have PI-derived motif and paleoAP3 motif (which specifically belong to *paleoAP3* gene). Both the PI-derived motif and euAP3 motif are found in the C-terminal domain of the *euAP3* gene, while the PI-derived motif is only found in the C-terminal domain of the *PI* gene. In carnation, *DcaMADS27* and *DcaMADS28* are members of the PI evolution line; *DcaMADS29* and *DcaMADS30* belongs to the *euAP3* evolution line and *DcaMADS31* belongs to *TM6* (Table S6 and Figure S4). The class B subfamily genes in carnation were involved in two duplication events that contributed to the three evolution lines (*PI* evolution line, *euAP3* evolution line, and *TM6*), which may cause these genes to function differently. Previous study showed that the class B subfamily genes were conserved in determining petal and stamen characteristics, but different evolutionary lines had dynamic changes in time and space [92]. There are three evolution lines in carnation: the *PI* evolution line and *euAP3* evolution line have two genes, which have different expression, and these genes may play different roles in stamen development in carnation. Our results provide the basis for studying the functional differentiation of carnation class B genes.

*SEP* subfamily: Phylogenetic evolution analysis showed that *SEP* subfamily genes experienced multiple gene duplication events during their evolution. The first duplication event generated SEP3 (*SEP3* was previously named *AGL9*, and *SEP1/SEP2/SEP4* were formerly named *AGL2/AGL4/AGL3*). The evolution lines of *SEP1/SEP2/SEP4* underwent two gene duplication events, producing *SEP1/SEP2, FBP9/FBP23, SEP4* evolution. This study revealed that *DcaMADS1* belongs to *SEP1; DcaMADS2* and *DcaMADS3* are members of the *SEP3* evolution line; *DcaMADS6*, *DcaMADS7*, and *DcaMADS8* belongs to *SEP4* (Table S6 and Figure S5). There are some reports about E-functional genes expressed in different tissues of various species such as *Arabidopsis* [93], the petunia *FBP2* gene [55,94], the *TM5* gene [95], the *LeMADSRIN* gene [96], and the *Gerbera hybrida GRCA1/2* gene. The number of *SEP* subfamily genes, their expression pattern and functions vary with the species and so these genes may also have different functions in carnation. What the functions of these genes of *SEP* subfamily in carnation are requires further study.

### 4.2. A Model in Flower Organ Identity of Carnation

**Figure 8:**
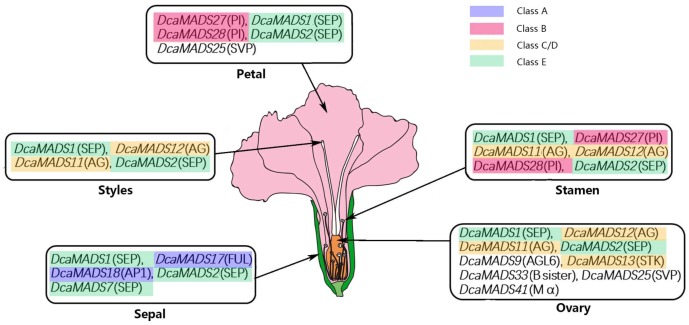
The high expression pattern of the *DcaMADSs* gene in carnation. The genes in rectangular boxes point to their expression being strongly detected, and the amount of expression decreased in turn.

Transcription factors of MADS-box genes play specific roles in flower organ development, especially in specifying floral organ identity, which have been revealed in the model eudicots *Arabidopsis* [6,97] and *Antirrhinum* [25]. Different subfamilies have different expression patterns. In this study, we investigated MADS-box in carnation, which can regulate flower development and different organ formation. Some genes in every subfamily have extremely low, or no, transcript abundances in flower organ tissues, while other subfamily genes display diverse expression patterns representing the distinct roles of the different groups. Gene functions of A-, B-, C-, D-, and E classes in carnation showed similarities and differences to that in *Arabidopsis* and other species. In order to further clarify the function of these genes, we conducted an in-depth study of the expression of these genes in different flower organs of carnation. All genes were recalculated for expression with the same gene of lower expression as a reference (Figure S6) and these genes with relative expressions more than 30 were selected. A model of gene expression patterns in carnation (Figure 8) is proposed based on the well-known ABC model of *Arabidopsis*.

In the sepal of carnation, five genes (three class E genes (*DcaMADS1*, *DcaMADS2*, and *DcaMADS7*), two class A genes (*DcaMADS17* and *DcaMADS18*)) were strongly expressed, suggesting that these genes might function together to control the sepal in carnation flowers (Figure 8), in accordance with the *AP1/FUL* gene and *SEP* gene together involved in regulating the development of sepals and petals of *Arabidopsis* [70]. Based on these results, we proposed several questions which need a further study: Why were three E genes in sepals detected? Is the complex regulating sepal development the combination of four proteins together?

In petals of carnation, five genes (two class B genes (*DcaMADS27* and *DcaMADS28*), two class E genes (*DcaMADS1* and *DcaMADS2*), and one SVP gene (*DcaMADS25*)), strongly expressed, play an important role in carnation (Figure 8). A complex of one AP1, one SEP protein, and two class B proteins (AP3 and PI) determines petal identity in the floral quartet model of *Arabidopsis* [98]. Compared with *Arabidopsis*, why are class A genes not strongly detected in the petals of carnation? This phenomenon may be due to the special structure of carnation or there may be other genes undetected that are functioning in the petal.

In the stamen of carnation, two B class (*DcaMADS27* and *DcaMADS28*), two C class (*DcaMADS11* and *DcaMADS12*), and two E class (*DcaMADS1* and *DcaMADS2*) genes were strongly expressed (Figure 8), while previous studies show that a complex of one SEP, one AP3, one PI protein, and one AG protein determines stamen identity in *Arabidopsis* [14]. Two genes have been added in carnation compared to the classic quarter model and what is the function of these two extra genes? We speculated that two B class or two C class genes create a functional redundancy or these six genes can form new complexes involved in the regulation of the stamen in carnation. We also found *DcaMAD31* (TM6) of class B genes in carnation were expressed in sepal, petals, and stamens, and previous studies found that TM6 plays a vital role in regulating stamen identity [99], *DcaMAD31* did not present obviously in this trend, which may be due to it not playing a role in stamen development. These hypotheses require more experiments to be performed.

In the carpel of carnation, four genes (two class C genes (*DcaMADS11* and *DcaMADS12*), two class E genes (*DcaMADS1* and *DcaMADS2*)), strongly expressed, play an important role in styles (Figure 8), and two class E genes, two class C genes, one AGL6, one STK, one B sister, one SVP, and one Mα were detected in ovaries suggesting their involvement in floral carpel development. Previous studies found that the combination of SEP genes with C-class and E-class genes regulated the development of the carpel [99]. This study showed that not only *DcaMADS13*(STK), but also *DcaMADS9* (AGL6), *DcaMADS33* (B sister), *DcaMADS41* (Mα), and *DcaMADS25* (SVP), were detected strongly in ovaries (Figure 8). These genes may be play an important role in floral organ identity.

Carnation is more diverse and complex than the simple *Arabidopsis* flower. Many of *DcaMADS* genes in different tissues exhibit expression patterns similar to those in *Arabidopsis* and other plant species, whereas other genes have their unique expression profiles. This difference may be related to difference species or the genetic evolution over a long history. The real reason requires us to make a deep exploration.

## Figures and Tables

**Table 1:** The complete list of the carnation MADS-box genes.

Name	Accession Number	ORF (bp)	Group	Clades
*DcaMADS1*	Dca58524.1	738	MIKC^c^	SEP
*DcaMADS2*	Dca4751.1	759	MIKC^c^	SEP
*DcaMADS3*	Dca45289.1	735	MIKC^c^	SEP
*DcaMADS4*	Dca42307.1	735	MIKC^c^	SEP
*DcaMADS5*	Dca47184.1	735	MIKC^c^	SEP
*DcaMADS6*	Dca61854.1	630	MIKC^c^	SEP
*DcaMADS7*	Dca42755.1	753	MIKC^c^	SEP
*DcaMADS8*	Dca22562.1	438	MIKC^c^	SEP
*DcaMADS9*	Dca72.1	777	MIKC^c^	AGL6
*DcaMADS10*	Dca19158.1	540	MIKC^c^	
*DcaMADS11*	Dca50159.1	750	MIKC^c^	AG/STK
*DcaMADS12*	Dca35398.1	756	MIKC^c^	AG/STK
*DcaMADS13*	Dca13518.1	699	MIKC^c^	AG/STK
*DcaMADS14*	Dca73.1	663	MIKC^c^	SOC1
*DcaMADS15*	Dca19159.1	666	MIKC^c^	SOC1
*DcaMADS16*	Dca23368.1	648	MIKC^c^	SOC1
*DcaMADS17*	Dca61853.1	765	MIKC^c^	AP1/FUL
*DcaMADS18*	Dca59408.1	585	MIKC^c^	AP1/FUL
*DcaMADS19*	Dca21554.1	249	MIKC^c^	
*DcaMADS20*	Dca51118.1	339	MIKC^c^	AGL12
*DcaMADS21*	Dca27570.1	309	MIKC^c^	AGL17
*DcaMADS22*	Dca58547.1	756	MIKC^c^	AGL15
*DcaMADS23*	Dca57893.1	726	MIKC^c^	AGL15
*DcaMADS24*	Dca7325.1	666	MIKC^c^	SVP
*DcaMADS25*	Dca5233.1	1935	MIKC^c^	SVP
*DcaMADS26*	Dca56094.1	600	MIKC^c^	SVP
*DcaMADS27*	Dca17660.1	642	MIKC^c^	AP3/PI
*DcaMADS28*	Dca7718.1	456	MIKC^c^	AP3/PI
*DcaMADS29*	Dca35875.1	543	MIKC^c^	AP3/PI
*DcaMADS30*	Dca24326.1	699	MIKC^c^	AP3/PI
*DcaMADS31*	Dca28764.1	645	MIKC^c^	AP3/PI
*DcaMADS32*	Dca52384.1	672	MIKC^c^	B sister
*DcaMADS33*	Dca35154.1	615	MIKC^c^	B sister
*DcaMADS34*	Dca45290.1	813	MIKC^c^	FLC
*DcaMADS35*	Dca42306.1	747	MIKC^c^	
*DcaMADS36*	Dca62484.1	792		
*DcaMADS37*	Dca41798.1	903	MIKC*	MIKC*
*DcaMADS38*	Dca21633.1	1074	MIKC*	MIKC*
*DcaMADS39*	Dca37955.1	1050		
*DcaMADS40*	Dca20695.1	747	Mα	Mα
*DcaMADS41*	Dca37633.1	1641	Mα	Mα
*DcaMADS42*	Dca46738.1	669	Mγ	Mγ
*DcaMADS43*	Dca21085.1	1107	Mγ	Mγ
*DcaMADS44*	Dca21084.1	696	Mγ	Mγ
*DcaMADS45*	Dca50134.1	474	Mγ	Mγ
*DcaMADS46*	Dca38557.1	687	Mγ	Mγ

ORF: open reading frames; SEP: SEPALLATA; AGL6: AGAMOUS-LIKE 6; AG: AGAMOUS; STK: SEEDSTICK; SOC1: SUPPRESSOR OF OVEREXPRESSION OF CO1; AP1:APETALA1; FUL: FRUITFULL; AGL12: AGAMOUS-LIKE 12; AGL15: AGAMOUS-LIKE 15; SVP: SHORT VEGETATIVE PHASE; AP3: APETALA3; PI: PISTILLATA; FLC: FLOWERING LOCUS C.

## Supplementary Materials

The following are available online at http://www.mdpi.com/2073-4425/9/4/193/s1, Table S1: *Arabidopsis* MADS sequences for the phylogenetic tree, Table S2: Rice MADS sequences for the phylogenetic tree, Table S3: Primers for quantitative PCR of *DcaMADSs*, Table S4: Primers for subcellular localization of DcaMADSs, Table S5: The number of B and E class genes in different species, Table S6: Sequences of B and E class genes for the phylogenetic tree, Figure S1: The ORF of DcaMADS6, DcaMADS8, DcaMADS18, DcaMADS28, and DcaMADS29, Figure S2: Conserved motifs of 39 carnation MADS-box proteins identified using the MEME program. The information of Motifs 1 to 15, Figure S3: The phylogenetic tree of the 39 *DcaMADS* genes was generated by the neighbour-joining (NJ) algorithm using Molecular Evolutionary Genetics Analysis (MEGA version 6.0) software. The subgroups are marked in different colors. (a) Phylogenetic tree of *D. caryophyllus* and Rice type II proteins. (b) Phylogenetic tree of Rice and *D. caryophyllus* type I proteins, Figure S4: The phylogenetic tree of the class B genes in carnaiton was generated by the neighbour-joining (NJ) algorithm using MEGA (version 6.0) software, Figure S5: The phylogenetic tree of the class E genes in carnaiton was generated by the neighbour-joining (NJ) algorithm using MEGA (version 6.0) software, Figure S6: Organ specific expression of 35 *DcaMADS* genes at different flower whorls.

## References

[1] Norman C., Runswick M., Pollock R., Treisman R. (1988). Isolation and properties of cDNA clones encoding SRF, a transcription factor that binds to the c-*fos* serum response element. Cell.

[2] Pellegrini L., Tan S., Richmond T.J. (1995). Structure of serum response factor core bound to DNA. Nature.

[3] Shore P., Sharrocks A.D. (1995). The MADS-box family of transcription factors. FEBS J..

[4] Sasaki K., Aida R., Yamaguchi H., Shikata M., Niki T., Nishijima T., Ohtsubo N. (2010). Functional divergence within class B MADS-box genes *TfGLO* and *TfDEF* in *Torenia fournieri* Lind. Mol. Genet. Genom..

[5] Alvarezbuylla E.R., Liljegren S.J., Pelaz S., Gold S.E., Burgeff C., Ditta G.S., Vergarasilva F., Yanofsky M.F. (2000). MADS-box gene evolution beyond flowers: Expression in pollen, endosperm, guard cells, roots and trichomes. Plant J. Cell Mol. Biol..

[6] Liu Y., Cui S., Wu F., Yan S., Lin X., Du X., Chong K., Schilling S., Theißen G., Meng Z. (2013). Functional conservation of MIKC*-Type MADS box genes in *Arabidopsis* and rice pollen maturation. Plant Cell.

[7] Parenicová L., De F.S., Kieffer M., Horner D.S., Favalli C., Busscher J., Cook H.E., Ingram R.M., Kater M.M., Davies B. (2003). Molecular and Phylogenetic Analyses of the Complete MADS-Box Transcription Factor Family in Arabidopsis: New Openings to the MADS World. Plant Cell.

[8] Gramzow L., Theißen G. (2013). Phylogenomics of MADS-Box genes in plants—Two opposing life styles in one gene family. Biology.

[9] Henschel K., Kofuji R., Hasebe M., Saedler H., Münster T., Theissen G. (2002). Two ancient classes of MIKC-type MADS-box genes are present in the moss *Physcomitrella patens*. Mol. Biol. Evol..

[10] Bodt S.D., Raes J., Florquin K., Rombauts S., Rouzé P., Theißen G., Peer Y.V.D. (2003). Genomewide structural annotation and evolutionary analysis of the type I MADS-Box genes in plants. J. Mol. Evol..

[11] Wells C.E., Vendramin E., Jimenez T.S., Verde I., Bielenberg D.G. (2015). A genome-wide analysis of MADS-box genes in peach [*Prunus persica* (L.) Batsch]. BMC Plant Biol..

[12] Kaufmann K., Melzer R., Theissen G. (2005). MIKC-type MADS-domain proteins: Structural modularity, protein interactions and network evolution in land plants. Gene.

[13] Smaczniak C., Immink R.G., Angenent G.C., Kaufmann K. (2012). Developmental and evolutionary diversity of plant MADS-domain factors: Insights from recent studies. Development.

[14] Coen E.S., Meyerowitz E.M. (1991). The war of the whorls: Genetic interactions controlling flower development. Nature.

[15] Moon J., Suh S.S., Lee H., Choi K.R., Hong C.B., Paek N.C., Kim S.G., Lee I. (2003). The *SOC1* MADS-box gene integrates vernalization and gibberellin signals for flowering in *Arabidopsis*. Plant J..

[16] Lee J.H., Yoo S.J., Park S.H., Hwang I., Lee J.S., Ahn J.H. (2007). Role of *SVP* in the control of flowering time by ambient temperature in *Arabidopsis*. Genes Dev..

[17] Liu C., Chen H., Er H.L., Soo H.M., Kumar P.P., Han J.H., Liou Y.C., Yu H. (2008). Direct interaction of *AGL24* and *SOC1* integrates flowering signals in *Arabidopsis*. Development.

[18] Hu J.Y., Meaux J.D. (2014). *miR824*-regulated AGAMOUS-LIKE16 contributes to flowering time repression in *Arabidopsis*. Plant Cell.

[19] Adamczyk B.J., Lehti-Shiu M.D., Fernandez D.E. (2007). The MADS domain factors AGL15 and AGL18 act redundantly as repressors of the floral transition in Arabidopsis. Plant J..

[20] Liljegren S.J., Ditta G.S., Eshed Y., Savidge B., Bowman J.L., Yanofsky M.F. (2000). SHATTERPROOF MADS-box genes control seed dispersal in Arabidopsis. Nature.

[21] Tapialópez R., Garcíaponce B., Dubrovsky J.G., Garayarroyo A., Pérezruíz R.V., Kim S.H., Acevedo F., Pelaz S., Alvarezbuylla E.R. (2008). An *AGAMOUS*-related MADS-box gene, *XAL1* (*AGL_12_*), regulates root meristem cell proliferation and flowering transition in Arabidopsis. Plant Physiol..

[22] Theissen G., Saedler H. (2001). Plant biology. Floral quartets. Nature.

[23] Ratcliffe O.J., Kumimoto R.W., Wong B.J., Riechmann J.L. (2003). Analysis of the Arabidopsis *MADS AFFECTING FLOWERING* gene family: *MAF2* prevents vernalization by short periods of cold. Plant Cell.

[24] Michaels S.D., Amasino R.M. (1999). *FLOWERING LOCUS C* encodes a novel MADS domain protein that acts as a repressor of flowering. Plant Cell.

[25] Rounsley S.D., Ditta G.S., Yanofsky M.F. (1995). Diverse roles for MADS box genes in Arabidopsis development. Plant Cell.

[26] Heuer S., Lörz H., Dresselhaus T. (2000). The MADS box gene *ZmMADS2* is specifically expressed in maize pollen and during maize pollen tube growth. Plant Reprod..

[27] Zobell O., Faigl W., Saedler H., Münster T. (2010). MIKC* MADS-box proteins: Conserved regulators of the gametophytic generation of land plants. Mol. Biol. Evol..

[28] Masiero S., Colombo L., Grini P.E., Schnittger A., Kater M.M. (2011). The emerging importance of type I MADS box transcription factors for plant reproduction. Plant Cell.

[29] Barker E.I., Ashton N.W. (2013). A parsimonious model of lineage-specific expansion of MADS-box genes in *Physcomitrella patens*. Plant Cell Rep..

[30] Yagi M. (2015). Recent progress in genomic analysis of ornamental plants, with a focus on Carnation. Hortic. J..

[31] Hileman L.C., Sundstrom J.F., Litt A., Chen M., Shumba T., Irish V.F. (2006). Molecular and phylogenetic analyses of the MADS-box gene family in tomato. Mol. Biol. Evol..

[32] Arora R., Agarwal P., Ray S., Singh A.K., Singh V.P., Tyagi A.K., Kapoor S. (2007). MADS-box gene family in rice: Genome-wide identification, organization and expression profiling during reproductive development and stress. BMC Genom..

[33] Zhao Y., Li X., Chen W., Peng X., Cheng X., Zhu S., Cheng B. (2011). Whole-genome survey and characterization of MADS-box gene family in maize and sorghum. Plant Cell Tissue Organ Cult..

[34] Gan D.F. (2012). Genome-wide sequence characterization analysis of MADS-Box transcription factor gene family in cucumber (*Cucumis sativus* L.). J. Nucl. Agric. Sci..

[35] Shu Y., Yu D., Wang D., Guo D., Guo C. (2013). Genome-wide survey and expression analysis of the MADS-box gene family in soybean. Mol. Biol. Rep..

[36] Duan W., Song X., Liu T., Huang Z., Ren J., Hou X., Li Y. (2015). Genome-wide analysis of the MADS-box gene family in *Brassica rapa* (Chinese cabbage). Mol. Genet. Genom..

[37] Wei X., Wang L., Yu J., Zhang Y., Li D., Zhang X. (2015). Genome-wide identification and analysis of the MADS-box gene family in sesame. Gene.

[38] Li C., Wang Y., Xu L., Nie S., Chen Y., Liang D., Sun X., Karanja B.K., Luo X., Liu L. (2016). Genome-wide characterization of the MADS-Box gene family in Radish (*Raphanus sativus* L.) and assessment of its roles in flowering and floral organogenesis. Front. Plant Sci..

[39] Yagi M., Kosugi S., Hirakawa H., Ohmiya A., Tanase K., Harada T., Kishimoto K., Nakayama M., Ichimura K., Onozaki T. (2014). Sequence Analysis of the genome of Carnation (*Dianthus caryophyllus* L.). DNA Res..

[40] TAIR Website. http://www.arabidopsis.org/.

[41] The Rice Genome Annotation Project. http://rice.plantbiology.msu.edu/.

[42] The National Center for Biotechnology Information. https://www.ncbi.nlm.nih.gov/.

[43] SMART. http://smart.embl.de/.

[44] Finn R.D., Coggill P., Eberhardt R.Y., Eddy S.R., Mistry J., Mitchell A.L., Potter S.C., Punta M., Qureshi M., Sangrador-Vegas A. (2016). The Pfam protein families database: Towards a more sustainable future. Nucleic Acid Res..

[45] Bailey T.L., Boden M., Buske F.A., Frith M., Grant C.E., Clementi L., Ren J., Li W.W., Noble W.S. (2009). MEME SUITE: Tools for motif discovery and searching. Nucleic Acids Res..

[46] Carnation genome. http://carnation.kazusa.or.jp/.

[47] Larkin M.A., Blackshields G., Brown N.P., Chenna R., Mcgettigan P.A., Mcwilliam H., Valentin F., Wallace I.M., Wilm A., Lopez R. (2007). Clustal W and Clustal X version 2.0. Bioinformatics.

[48] Tamura K., Stecher G., Peterson D., Filipski A., Kumar S. (2013). MEGA6: Molecular Evolutionary Genetics Analysis version 6.0. Comput. Appl. Biosci. CABIOS.

[49] Yong J.L., Hwang I. (2001). Identification of a signal that distinguishes between the chloroplast outer envelope membrane and the endomembrane system in vivo. Plant Cell.

[50] Baudinette S.C., Stevenson T.W., Savin K.W. (2000). Isolation and characterisation of the carnation floral-specific MADS box gene, *CMB2*. Plant Sci..

[51] Tian Y., Dong Q., Ji Z., Chi F., Cong P., Zhou Z. (2015). Genome-wide identification and analysis of the MADS-box gene family in apple. Gene.

[52] Johansen B., Pedersen L.B., Skipper M., Frederiksen S. (2002). MADS-box gene evolution-structure and transcription patterns. Mol. Phylogenetics Evol..

[53] Bailey T.L., Elkan C. (1994). Fitting a mixture model by expectation maximization to discover motifs in biopolymers. Proc. Int. Conf. Intell. Syst. Mol. Biol..

[54] Gramzow L., Theissen G. (2010). A hitchhiker’s guide to the MADS world of plants. Genome Biol..

[55] Ferrario S., Immink R.G., Shchennikova A., Busscherlange J., Angenent G.C. (2003). The MADS box gene *FBP2* is required for *SEPALLATA* function in petunia. Plant Cell.

[56] Pelaz S., Ditta G.S., Baumann E., Wisman E., Yanofsky M.F. (2000). B and C floral organ identity functions require *SEPALLATA* MADS-box genes. Nature.

[57] Malcomber S.T., Kellogg E.A. (2005). *SEPALLATA* gene diversification: Brave new whorls. Trends Plant Sci.

[58] Castillejo C., Romera-Branchat M., Pelaz S. (2005). A new role of the Arabidopsis *SEPALLATA3* gene revealed by its constitutive expression. Plant J..

[59] Ohmori S., Kimizu M., Sugita M., Miyao A., Hirochika H., Uchida E., Nagato Y., Yoshida H. (2009). *MOSAIC FLORAL ORGANS1*, an *AGL6*-like MADS box gene, regulates floral organ identity and meristem fate in rice. Plant Cell.

[60] Hao X., Fu Y., Zhao W., Liu L., Bade R., Hasi A., Hao J. (2016). Genome-wide identification and analysis of the MADS-box gene family in Melon. J. Am. Soc. Horticult. Sci..

[61] Pnueli L., Hareven D., Rounsley S.D., Yanofsky M.F., Lifschitz E. (1994). Isolation of the tomato AGAMOUS gene TAG1 and analysis of its homeotic role in transgenic plants. Plant Cell.

[62] Ray A., Robinson-Beers K., Ray S., Baker S.C., Lang J.D., Preuss D., Milligan S.B., Gasser C.S. (1994). Arabidopsis floral homeotic gene BELL (BEL1) controls ovule development through negative regulation of AGAMOUS gene (AG). Proc. Natl. Acad. Sci. USA.

[63] Favaro R., Pinyopich A., Battaglia R., Kooiker M., Borghi L., Ditta G., Yanofsky M.F., Kater M.M., Colombo L. (2003). MADS-box protein complexes control carpel and ovule development in Arabidopsis. Plant Cell.

[64] Lee S., Kim J., Han J.J., Han M.J., An G. (2004). Functional analyses of the flowering time gene *OsMADS50*, the putative *SUPPRESSOR OF OVEREXPRESSION OF CO 1/AGAMOUS-LIKE 20 (SOC1/AGL20)* ortholog in rice. Plant J..

[65] Lee J. (2010). Regulation and function of SOC1, a flowering pathway integrator. J. Exp. Bot..

[66] Kobayashi K., Yasuno N., Sato Y., Yoda M., Yamazaki R., Kimizu M., Yoshida H., Nagamura Y., Kyozuka J. (2012). Inflorescence meristem identity in rice is specified by overlapping functions of three *AP1/FUL*-like MADS box genes and *PAP2*, a *SEPALLATA* MADS box gene. Plant Cell.

[67] Ahn M.S., Kim Y.S., Han J.Y., Yoon E.S., Yong E.C. (2015). Panax ginseng *PgMADS1*, an AP1/FUL-like MADS-box gene, is activated by hormones and is involved in inflorescence growth. Plant Cell Tissue Organ Cult..

[68] Becker A., Theißen G. (2003). The major clades of MADS-box genes and their role in the development and evolution of flowering plants. Mol. Phylogenetics Evol..

[69] Litt A. (2007). An evaluation of A-function: Evidence from the *APETALA1* and *APETALA2* gene lineages. Int. J. Plant Sci..

[70] Gu Q., Ferrándiz C., Yanofsky M.F., Martienssen R. (1998). The FRUITFULL MADS-box gene mediates cell differentiation during Arabidopsis fruit development. Development.

[71] Burgeff C., Liljegren S.J., Tapia-López R., Yanofsky M.F., Alvarez-Buylla E.R. (2002). MADS-box gene expression in lateral primordia, meristems and differentiated tissues of *Arabidopsis thaliana* roots. Planta.

[72] Zachgo S., Saedler H., Schwarz-Sommer Z. (1997). Pollen-specific expression of *DEFH125*, a MADS-box transcription factor in *Antirrhinum* with unusual features. Plant J. Cell Mol. Biol..

[73] Mouradov A., Hamdorf B., Teasdale R.D., Kim J.T., Winter K.U., Theißen G. (1999). A DEF/GLO-like MADS-box gene from a gymnosperm: Pinus radiata contains an ortholog of angiosperm B class floral homeotic genes. Genesis.

[74] Whipple C.J., Ciceri P., Padilla C.M., Ambrose B.A., Bandong S.L., Schmidt R.J. (2004). Conservation of B-class floral homeotic gene function between maize and *Arabidopsis*. Development.

[75] Becker A., Kaufmann K., Freialdenhoven A., Vincent C., Li M.A., Saedler H., Theissen G. (2002). A novel MADS-box gene subfamily with a sister-group relationship to class B floral homeotic genes. Mol. Genet. Genom..

[76] De Folter S., Shchennikova A.V., Franken J., Busscher M., Baskar R., Grossniklaus U., Angenent G.C., Immink R.G.H. (2006). A B_sister_ MADS-box gene involved in ovule and seed development in petunia and Arabidopsis. Plant J..

[77] Prasad K., Zhang X., Tobón E., Ambrose B.A. (2010). The Arabidopsis B-sister MADS-box protein, GORDITA, represses fruit growth and contributes to integument development. Plant J..

[78] Erdmann R., Gramzow L., Melzer R., Theissen G., Becker A. (2010). *GORDITA* (AGL63) is a young paralog of the *Arabidopsis thaliana* B_sister_ MADS box gene *ABS* (*TT16*) that has undergone neofunctionalization. Plant J..

[79] Michaels S.D., Amasino R.M. (2001). Loss of *FLOWERING LOCUS C* activity eliminates the late-flowering phenotype of *FRIGIDA* and autonomous pathway mutations but not responsiveness to vernalization. Plant Cell.

[80] Michaels S.D., He Y., Scortecci K.C., Amasino R.M. (2003). Attenuation of *FLOWERING LOCUS C* activity as a mechanism for the evolution of summer-annual flowering behavior in Arabidopsis. Proc. Natl. Acad. Sci. USA.

[81] Leseberg C.H., Li A., Kang H., Duvall M., Mao L. (2006). Genome-wide analysis of the MADS-box gene family in *Populus trichocarpa*. Gene.

[82] Adamczyk B.J., Fernandez D.E. (2009). MIKC* MADS domain heterodimers are required for pollen maturation and tube growth in Arabidopsis. Plant Physiol..

[83] Verelst W., Saedler H., Münster T. (2007). MIKC* MADS-protein complexes bind motifs enriched in the proximal region of late pollen-specific Arabidopsis promoters. Plant Physiol..

[84] Shih M.C., Chou M.L., Yue J.J., Hsu C.T., Chang W.J., Ko S.S., Liao D.C., Huang Y.T., Chen J.J., Yuan J.L. (2014). BeMADS1 is a key to delivery MADSs into nucleus in reproductive tissues-*De novo* characterization of *Bambusa edulis* transcriptome and study of MADS genes in bamboo floral development. BMC Plant Biol..

[85] Díazriquelme J., Lijavetzky D., Martínezzapater J.M., Carmona M.J. (2008). Genome-wide analysis of MIKC-type MADS box genes in grapevine. Plant Physiol..

[86] Xu Z., Zhang Q., Sun L., Du D., Cheng T., Pan H., Yang W., Wang J. (2014). Genome-wide identification, characterisation and expression analysis of the MADS-box gene family in *Prunus mume*. Mol. Genet. Genom..

[87] Lin C.S., Hsu C.T., Liao C., Chang W.J., Chou M.L., Huang Y.T., Chen J.J., Ko S.S., Chan M.T., Shih M.C. (2015). Transcriptome-wide analysis of the MADS-box gene family in the orchid *Erycina pusilla*. Plant Biotechnol. J..

[88] Zahn L.M. (2005). To B or Not to B a Flower: The Role of *DEFICIENS* and *GLOBOSA* orthologs in the evolution of the angiosperms. J. Hered..

[89] Aoki S., Uehara K., Imafuku M., Hasebe M., Ito M. (2004). Phylogeny and divergence of basal angiosperms inferred from *APETALA3*- and *PISTILLATA*-like MADS-box genes. J. Plant Res..

[90] Kramer E.M., Dorit R.L., Irish V.F. (1998). Molecular Evolution of Genes Controlling Petal and Stamen Development: Duplication and Divergence within the *APETALA3* and *PISTILLATA* MADS-Box Gene Lineages. Genetics.

[91] Kramer E.M., Su H.J., Wu C.C., Hu J.M. (2006). A simplified explanation for the frameshift mutation that created a novel C-terminal motif in the *APETALA3* gene lineage. BMC Evol. Biol..

[92] Jaramillo M.A., Kramer E.M. (2007). Molecular evolution of the petal and stamen identity genes, *APETALA3* and *PISTILLATA*, after petal loss in the Piperales. Mol. Phylogenetics Evol..

[93] Biewers S.M. (2014). *Sepallata* Genes and Their Role during Floral Organ Formation. Ph.D. Thesis.

[94] Angenent G.C., Franken J., Busscher M., Weiss D., van Tunen A.J. (1994). Co-suppression of the petunia homeotic gene *FBP2* affects the identity of the generative meristem. Plant J..

[95] Pnueli L., Hareven D., Broday L., Hurwitz C., Lifschitz E. (1994). The TM5 MADS Box gene mediates organ differentiation in the three inner whorls of tomato flowers. Plant Cell.

[96] Vrebalov J., Ruezinsky D., Padmanabhan V., White R., Medrano D., Drake R., Schuch W., Giovannoni J. (2002). A MADS-Box Gene necessary for fruit ripening at the tomato ripening-inhibitor (Rin) locus. Science.

[97] Bemer M., Heijmans K., Airoldi C., Davies B., Angenent G.C. (2010). An atlas of type I MADS box gene expression during female gametophyte and seed development in Arabidopsis. Plant Physiol..

[98] Theißen G., Melzer R., Rümpler F. (2016). MADS-domain transcription factors and the floral quartet model of flower development: Linking plant development and evolution. Development.

[99] Rijpkema A.S., Royaert S., Zethof J., Van d.W.G., Gerats T., Vandenbussche M. (2006). Analysis of the Petunia *TM6* MADS box gene reveals functional divergence within the *DEF/AP3* lineage. Plant Cell.

