# Supplementary material for: Identification and Characterization of the MADS-Box Genes and Their Contribution to Flower Organ in Carnation (Dianthus caryophyllus L.)
Source: Genes (Basel). 2018 Apr 4;9(4):193. doi: 10.3390/genes9040193 (PMC5924535; doi:10.3390/genes9040193)
Supplement: Supplementary file 1 [file genes-09-00193-s001.pdf]

Table S1. *Arabidopsis* MADS sequences for the phylogenetic tree.

Gene name	AGI/GenBank	Phylogenetic group
PISTILLATA	At5g20240	MIKC
APETALA3	At3g54340	MIKC
AGAMOUS	At4g18960	MIKC
AGL1=SHP1	At3g58780	MIKC
AGL2=SEP1	At5g15800	MIKC
AGL3=SEP4	At2g03710	MIKC
AGL4=SEP2	At3g02310	MIKC
AGL5=SHP2	At2g42830	MIKC
AGL6	At2g45650	MIKC
AGL7=AP1	At1g69120	MIKC
AGL8=FUL	At5g60910	MIKC
AGL9=SEP3	At1g24260	MIKC
AGL10=CAL	At1g26310	MIKC
AGL11 STK	At4g09960	MIKC
AGL12	At1g71692	MIKC
AGL13	At3g61120	MIKC
AGL14	At4g11880	MIKC
AGL15	At5g13790	MIKC
AGL16	At3g57230	MIKC
AGL17	At2g22630	MIKC
AGL18	At3g57390	MIKC
AGL19	At4g22950	MIKC
AGL20=SOC1	At2g45660	MIKC
AGL21	At4g37940	MIKC
AGL22=SVP	At2g22540	MIKC
AGL24	At4g24540	MIKC
AGL25=FLC	At5g10140	MIKC
AGL27=FLM	At1g77080	MIKC
AGL31	At5g65050	MIKC
AGL32=TT16	At5g23260	MIKC
AGL42	At5g62165	MIKC
AGL44=ANR1	At2g14210	MIKC
AGL63	At1g31140	MIKC
AGL68	At5g65080	MIKC
AGL69	At5g65070	MIKC
AGL70	At5g65060	MIKC
AGL71	At5g51870	MIKC
AGL72	At5g51860	MIKC
AGL79	At3g30260	MIKC
AGL30	At2g03060	Mδ
AGL65	At1g18750	Mδ
AGL66	At1g77980	Mδ
AGL67	At1g77950	Mδ
AGL94	At1g69540	Mδ
AGL104	At1g22130	Mδ
AGL33	At2g26320	Mδ
AGL23	At1g65360	Mα
AGL28	At1g01530	Mα
AGL29	At2g34440	Mα
AGL39	At5g27130	Mα
AGL40	At4g36590	Mα

AGL55	At1g60920	Mα
AGL56	At1g60880	Mα
AGL57	At3g04100	Mα
AGL58	At1g28450	Mα
AGL59	At1g28460	Mα
AGL60	At1g72350	Mα
AGL61	At2g24840	Mα
AGL62	At5g60440	Mα
AGL73	At5g38620	Mα
AGL74	At1g48150	Mα
AGL83	At5g49490	Mα
AGL84	At5g49420	Mα
AGL85	At1g54760	Mα
AGL91	At3g66656	Mα
AGL97	At1g46408	Mα
AGL99	At5g04640	Mα
AGL100	At1g17310	Mα
AGL102	At1g47760	Mα
AGL43	At5g40220	Mβ
AGL47	At5g55690	Mβ
AGL49	At1g60040	Mβ
AGL50	At1g59810	Mβ
AGL52	At4g11250	Mβ
AGL53	At5g27070	Mβ
AGL54	At5g27090	Mβ
AGL75	At5g41200	Mβ
AGL76	At5g40120	Mβ
AGL77	At5g38740	Mβ
AGL78	At5g65330	Mβ
AGL81	At5g39750	Mβ
AGL82	At5g58890	Mβ
AGL89	At5g27580	Mβ
AGL101[c]	At5g27050	Mβ
AGL93	At5g26950	Mβ
AGL98	At5g39810	Mβ
AGL103	At3g18650	Mβ
AGL36	At5g26645	Mγ
AGL37	At1g65330	Mγ
AGL38	At1g65300	Mγ
AGL41	At2g26880	Mγ
AGL45	At3g05860	Mγ
AGL46	At2g28700	Mγ
AGL48	At2g40210	Mγ
AGL80	At5g48670	Mγ
AGL86	At1g31630	Mγ
AGL87	At1g22590	Mγ
AGL90	At5g27960	Mγ
AGL92[b]	At1g31640	Mγ
AGL95	At2g15660	Mγ
AGL96	At5g06500	Mγ

Table S2. Rice MADS sequences for the phylogenetic tree.

Name	Rigr	ORF(bp)	Length	Introns	Type
OsMADS1	LOC_Os03g11614	774	257	7	MIKC^c
OsMADS2	LOC_Os01g66030	630	209	6	MIKC^c
OsMADS3	LOC_Os01g10504	864	287	8	MIKC^c
OsMADS4	LOC_Os05g34940	633	210	6	MIKC^c
OsMADS5	LOC_Os06g06750	678	225	7	MIKC^c
OsMADS6	LOC_Os02g45770	753	250	7	MIKC^c
OsMADS7/45	LOC_Os08g41950	933	310	9	MIKC^c
OsMADS8/24	LOC_Os09g32948	747	248	7	MIKC^c
OsMADS13	LOC_Os12g10540	813	270	7	MIKC^c
OsMADS15	LOC_Os07g01820	804	267	7	MIKC^c
OsMADS16	LOC_Os06g49840	675	224	6	MIKC^c
OsMADS17	LOC_Os04g49150	765	254	7	MIKC^c
OsMADS18/28	LOC_Os07g41370	750	249	7	MIKC^c
OsMADS20	LOC_Os12g31748	522	233	5	MIKC^c
OsMADS21	LOC_Os01g66290	798	265	7	MIKC^c
OsMADS22	LOC_Os02g52340	687	228	7	MIKC^c
OsMADS23	LOC_Os08g33488	480	159	4	MIKC^c
OsMADS25	LOC_Os04g23910	684	227	7	MIKC^c
OsMADS26	LOC_Os08g02070	669	222	6	MIKC^c
OsMADS27	LOC_Os02g36924	723	240	7	MIKC^c
OsMADS29	LOC_Os02g07430	783	260	7	MIKC^c
OsMADS30	LOC_Os06g45650	666	221	7	MIKC^c
OsMADS31	LOC_Os04g52410	637	178	3	MIKC^c
OsMADS32	LOC_Os01g52680	591	196	6	MIKC^c
OsMADS33	LOC_Os12g10520	609	202	6	MIKC^c
OsMADS34	LOC_Os03g54170	720	239	7	MIKC^c
OsMADS37	LOC_Os08g41960	612	203	3	MIKC^c
OsMADS47	LOC_Os03g08754	753	250	7	MIKC^c
OsMADS50	LOC_Os03g03100	351	116	6	MIKC^c
OsMADS55	LOC_Os06g11330	672	223	7	MIKC^c
OsMADS56	LOC_Os10g39130	702	233	7	MIKC^c
OsMADS57	LOC_Os02g49840	726	241	7	MIKC^c
OsMADS58	LOC_Os05g11414	702	233	8	MIKC^c
OsMADS59	LOC_Os06g23950	234	77	1	MIKC^c
OsMADS61	LOC_Os04g38770	300	99	2	MIKC^c
OsMADS62	LOC_Os08g38590	1014	337	8	MIKC*
OsMADS63	LOC_Os06g11970	1083	360	10	MIKC*
OsMADS65	LOC_Os01g69850	495	164	4	MIKC*
OsMADS66	LOC_Os05g11380	303	100	1	MIKC^c
OsMADS68	LOC_Os11g43740	1158	385	10	MIKC*
OsMADS64	LOC_Os04g31804	750	249	6	Mα
OsMADS70	LOC_Os05g23780	657	218	0	Mα
OsMADS71	LOC_Os06g22760	717	238	0	Mα
OsMADS72	LOC_Os03g14850	558	185	1	Mα
OsMADS73	LOC_Os12g21850	585	194	0	Mα
OsMADS74	LOC_Os12g21880	360	119	1	Mα
OsMADS75	LOC_Os06g30810	633	210	0	Mα

OsMADS76	LOC_Os06g30830	762	241	2	Mα
OsMADS77	LOC_Os09g02780	537	178	0	Mα
OsMADS78	LOC_Os09g02830	627	208	0	Mα
OsMADS79	LOC_Os01g74440	627	208	0	Mα
OsMADS80	LOC_Os02g06860	861	286	0	Mα
OsMADS81	LOC_Os04g24790	630	209	0	Mγ
OsMADS82	LOC_Os04g24800	630	209	0	Mγ
OsMADS83	LOC_Os04g24810	630	209	0	Mγ
OsMADS84	LOC_Os04g25870	630	209	0	Mγ
OsMADS85	LOC_Os04g25920	630	209	0	Mγ
OsMADS86	LOC_Os03g37670	765	254	4	Mγ
OsMADS87	LOC_Os03g38610	750	249	0	Mγ
OsMADS88	LOC_Os01g18420	723	240	0	Mγ
OsMADS89	LOC_Os01g18440	921	306	0	Mγ
OsMADS90	LOC_Os07g04170	1473	490	4	Mβ
OsMADS91	LOC_Os01g11510	1851	604	0	Mβ
OsMADS92	LOC_Os01g23750	1221	406	0	Mβ
OsMADS93	LOC_Os01g23760	1224	407	0	Mβ
OsMADS94	LOC_Os01g23770	972	323	2	Mβ
OsMADS95	LOC_Os01g23780	837	278	2	Mβ
OsMADS96	LOC_Os01g67890	1452	483	0	Mβ
OsMADS97	LOC_Os01g68420	819	272	0	Mβ
OsMADS98	LOC_Os01g68560	1440	479	0	Mβ
OsMADS99	LOC_Os04g25930	465	154	1	Mγ

Table S3. Primers for quantitative PCR of DcaMADSs.

Gene name	Forward primer (5'->3')	Reverse primer (5'->3')
DcaMADS1	GGTGAAAGATAGTCAAATGAGGCAG	GTCAATGGGTTGGAAAAGAGGTT
DcaMADS2	TGAGGTTGGTTATGGAAGGGAC	GTTCATACTTGGACCGTCTTGTG
DcaMADS3	ATCTTCGCAGTGGAACGGACAA	CCTGCTGATAACCGATTTGTAAG
DcaMADS6	TGGGAAAGTCAAGAACAACA	CAGCAATATCAACTTCACTTGG
DcaMADS7	CTACCCAATCTGAGGCATTTTTC	AGACCTGTTGCTGGCTTGTTGT
DcaMADS8	TCATTTCGTCCGAATTGGGA	GCGTTCATTTGATCTTGTGTTG
DcaMADS9	GTTTCAAGCCCGAACCCGC	ACCACCCTTGGTGAAGAAAA
DcaMADS11	GCATTAGTAGAATCCGATCCAA	TCGTAGTCACCACCACCCG
DcaMADS12	CGAATCCGATCCAAAAAGAATG	TCGTACTCGCTGCTACCTCC
DcaMADS13	GAAGTCGAAAATCTCCAGCAGC	GAAACTTCTTGTCAGTAGGAATGGT
DcaMADS14	TAAGGAGCCATCCGTAGA	CGGCAATCCGATGAATAA
DcaMADS15	TAACGAACAAATTAAACGGCTGAC	TAAATCGGTCTCTACGTCGTCGC
DcaMADS16	GCCTTCCTCGCTTGGATC	AACGGTTGTTTCTGCTTTCC
DcaMADS17	ACAATGGGAGCAACAAAC	CTACTGATCTGCGAGGTG
DcaMADS18	ACAAGGTGTTGGTGATGA	GTATTGGCGATTTGGCTC
DcaMADS20	CTTTAAGGCCGCTGAATC	TACCAAAGCCCTTTCTCA
DcaMADS21	CCATACTTTGCGATGCTC	TGCCTCCTTTGATTTCGT
DcaMADS22	TGCCGATGCAGCTTGTAG	GAAACGGTTGGACTTGCA
DcaMADS23	GCGTATTGATGAAGGGTG	CCATCTGACCGTTGGATT
DcaMADS24	CGAGGAATATCATCTACGAGG	CAGAATACGGTAATCCCAAC
DcaMADS25	GTGGCGAGTGTTCAGGAT	ACACGGGATTGACTTCCA
DcaMADS26	GGAAAGGACGCTCAACTCTC	TGTGTCCGATGGTAAGTTGT
DcaMADS27	GCACAAGCAGGAGATGGA	AAGGGTTGTGATGCTGATT
DcaMADS28	GGAGGAGAATAATCAGCTTGTA	GATGGAATCGGGTGCTGA
DcaMADS29	CTACAAAGAAAAAGGTGCGAAATGG	GCAATCTGAGAGCGAGCAAACGAG
DcaMADS30	AAACACCGAAAGACGAGGAAGC	GCATCCTCAGACCAACAACTCG
DcaMADS31	TCCGAGAAACTATGAGGCG	TGAAAGTCAAGTGATCGGGT
DcaMADS33	ATACGTGCTCGAAAGGAA	GGGAAGCGGTAAGGATAG
DcaMADS34	GGGGCGGAGGAAGATAGA	AAGACGATAACGGCAACG
DcaMADS37	ACCCTAAACGAATTGACC	CACGGTTGAGGTCTTGTG
DcaMADS38	ACAGTCCTGGGCAACTAA	ATCAATACCGAGCATAGC
DcaMADS40	CTTCTTTGCGACATTAGG	GTTGCATTCTCCACCATC
DcaMADS41	CCAGCCACTACCCTCACT	GTTGGACATCCGCTTCTT
DcaMADS44	TTAGGGCGTGAAGCTGAT	TGCTCGTCGTCCCGAACTG
DcaMADS45	GAAGTACATGCTAAGCCAGGAG	GGATAAAGCGGACCCAAC
DcaGAPDH	CGGAAAGTTGACTGGTATGGC	CATCCTCGGTGTAGCCCAAAAT

Table S4. Primers for subcellular localization of DcaMADSs.

Gene name	Forward primer (5'->3')	Reverse primer (5'->3')
DcaMADS2	CGCGGATCCATGGGGAGAGGAAGAGTAGAATTG	ACGCGTCGACCATTAACCAACCCGTCATGAAGT
DcaMADS3	CGCGGATCCAGTATGGGAAGAGGAAGAGTAGA	ACGCGTCGACCATTTGCATCCACCCTGGTAGA
DcaMADS7	TATCGAGCTCATGGGAAGAGGAAGAGTAGAACT	GCTCTAGAAAGCATCCACCCGTGGATGTT
DcaMADS8	TATCGAGCTCATGGGGAGAGGAAGAGTAGAGTT	GCTCTAGAAAGCATCCATCCTTGTGCAA
DcaMADS12	GGATCCATGGAATTTTCAAGCCAAATAACT	GTCGACAACAAGTTGAAGAGGTGTTTGGTC
DcaMADS14	GGATCCATGGTGAGAGGAAAGACCCAAT	GTCGACAGCGCTAATAACACGCTTAATCTTG
DcaMADS15	GGATCCATGGCTCGAGGGAAGACGCAATT	GTCGACATTAATGACACGCTTATTTTTGCTC
DcaMADS17	GGATCCATGGGGAGGGGTAGGGTTCAG	GTCGACCTGATCTGCGAGGTGATTGA
DcaMADS20	GGATCCATGGCAAGAGGAAAGGTGC	GTCGACCCAAAGCCCTTTCTCAAGTAACTTG
DcaMADS22	GGATCCATGGGAAGAGGGAAAATAGA	GTCGACGGGAGAAACGGTTGGACTTG
DcaMADS26	GGATCCATGGTGAGGCAAAGAATTC	GTCGACTATAGGAGGCCCAAGTCTAAGAAAT
DcaMADS27	GGATCCATGGGGAGAGGAAAAATAGAGAT	GTCGACCATGCGATCTTGGAGGTTT
DcaMADS28	GGATCCATGGGGAGAGGAAAAATAGA	GTCGACGAGCCGATCTTGGAGG
DcaMADS30	GGATCCATGGCAAAAAGAGGAAAGATAGAAA	GTCGACAAGCAATGCGTAAGTAGTAACACAA
DcaMADS31	GGATCCATGGGAAGAGGGAAGCTTGAGATAA	GTCGACAGTGATCGGGTGACGGCTTAAAGCA

Table S5. The number of B and E class genes in different species.

Species	Maize	Sorghum	Rice	*Arabidopsis*	*E.pusilla*	Tomato	Poplar	Grape	Cucumber	Peach	Carnation
AP3/PI	6	3	4	2	4	4	3	3	3	3	5
SEP	3	5	5	4	4	5	5	3	4	4	6

Table S6. Sequences of B and E class genes for the phylogenetic tree.

Gene name	Accession number
Arabidopsis thaliana SEP1	NM_001125758.2
Arabidopsis thaliana SEP2	NM_111098
Arabidopsis thaliana SEP3	NM_102272.4
Arabidopsis thaliana SEP4	NM_179599
Arabidopsis thaliana PI	At5g20240
Arabidopsis thaliana AP3	At3g54340
Antirrhinum majus DEF	CAA44629
Antirrhinum majus GLO	Q03378
Petunia fbp2	M91666
Petunia x hybrida FBP4	AF335234
Petunia x hybrida FBP5	AF335235
Petunia x hybrida FBP9	AF335236
Petunia x hybrida FBP23	AF335241
Petunia x hybrida PMADS12	AY370527
Petunia x hybrida FBP1	Q03488
Petunia x hybrida GLO1	AAS46018
Petunia x hybrida DEF	AAQ72510
Petunia x hybrida pMADS2	CAA49568
Petunia x hybrida phTM6	AAS46017
Solanum lycopersicum DEF	NP_001234077
Solanum lycopersicum TDR6(TM6)	NP_001311309
Solanum lycopersicum LeAP3	AF052868

> DcaMADS6

ATGGGAAGAGGAAGAGTAGAACTAAAGAGGATAGAAAACAAAATCAATAGACAAGTC
ACATTTGCAAAGAGAAGAAATGGTCTTCTTAAGAAAGCTTATGAACTTTCTATTCTTTG
TGATGCTGAGGTTGCCCTAATTATCTTCTCTAATCCTGGCAAACTCTATGAATTTTCAAG
CAATTCTAGTATGCTTAAAACCATTGAAAAGTACCAGAGATGTAGTTATGGTGCTTTGG
AAACCACTGACATTCTCAATAATTCACAGGCTAATTACCAAGAATATTTAAAGCTAAAG
GCTAGAGTGGAAGTTCTTCAGCAATCTCAGAGGAATTTGTTGGGTGAAGATTTAGCAC
CACTGACTATAAAAGACCTACAGCAATTGGAACAACAGCTTGAGATTTCTCTTAAGCA
AATAAGATCCACCAAGACCAGTCATTTGCTCGATCAGCTTAGAGATCTGCAAAAAAGG
GAACAAGCTCTAACTGAAAATAACAGAAACTTGATTAAGGAGTTGGAAGAAAGTAAC
AATAATCATCAATACTCTACAAGACTTGGATGGGAAAGTCAAGAACAACAAATTATTCC
TATTCAATCTGAGGCTTTTTTCCAACCTCTTAGTTGCAATCAACAACCATCACAAATAA
GCTACCAACATCCATTGAGCCCAAGTGAAGTTGATATTGCTGCGACGAGCCAACAAAT
CGCGAGGTTTAACATCCATGGATGGATGCTTTGA

> DcaMADS8

ATGGGGAGAGGAAGAGTAGAGTTGAAGAGAATAGAGAACAAAATAAATAGACAAGTA
ACATTTGCAAAGAGAAGAAATGGGTTGTTGAAGAAGGCTTATGAATTATCAGTTCTTTG
TGATGCTGAAGTTGCTCTTATTGTATTTTCTAACCGTGGTAAACTCTATGAGTTTTGTAG
CACTTCCTGCATGAACAAAACATTGGAGAGATATCAGAGATGCAGCTATGGTTCCCTTG
AAACAAGTCAACCTTCTAAAGAGACTGAGCAGAGTAGCTACCAGGAGTATCTTAAGCT
CAAAGCCAAAGTTGATGTCCTACAACGATCCCATAGGAATTTGCTAGGGGAGGACTTG
GGAGAGTTGAGCACAAAAGAGTTGGAACAGCTTGAGCATCAATTGGACAAGTCTTTG
AGGCAAATCAGGTCTATTAAGACCCAAAATATGCTCGATCAACTTGCTGATCTTCAAAA
GAAGGAAGAGATGCTACTTGAGTCTAACAGAGCTTTAAAAACGAAGTTGGAAGAAAG
TTGTGCATCATTTCGTCCGAATTGGGATGGCCGCCAACCGGGTGACGGTTTTTTCGAAC
CATTACCATGCAACAACAATCTTCAAATAGGGTACAATGAAGCAACACAAGATCAAAT
GAACGCAACAACTTCAACTCAAAATGGGCATGGATTTGCACAAGGATGGATGCTTTGA

> DcaMADS18

ATGGGGAGGGGTAGGGTGCAATTGAAGAGAATAGAGAACAAAATAAATAGGCAAGTG
ACCTTCTCAAAGAGGAGAAGTGGTCTTGTGAAAAAAGCAAATGAAATTTCAGTGCTTT
GTGATGCTGAAGTTGCTCTCATCATTTTCTCTCATCGGGGCAAACTCTTTGAGTTCTCCT
CCGACTCTTGCATGGAGAAGGTATTAGAAAGGTACGAGAGATACTCTTACGCCGAGAA
GCAACTAGCTTCAAATGACCCTGATACTCAGATTAATTGGACATTTGATTTTGCCAAAC
TCAAGGCTAAGCTTGATCTCCTACAAAGAAATCATAGGCAATACCTAGGTCAAGATTTG
GATACGCTCAACATGAAAGAGCTTCAAAGTTTAGAGCAACAACTTGATACCGGCCTCA
AACACATTAGATCAAGAAAGAATCAATTGATGCACGAGTCTATCTCTGAACTCCAGAA
GAAGGAGAGATCAATGCAAGAGCAAAACAACATGTTAGCAAAGAAGATCAAGGAGA
AAGAAAAAGTTACCGTGCAACAGCAGCAGCAGCAGCAGCAGCAACAACAATGGCAG
CAACAAAGTCATGAACAAGGTGTTGGTGATGACTCAAATTATCTAATGCACCCACCTCT
TCCTTCTTTGAACATCAGAAACTCATACGAAGGAGGAGGAGGAGGAGAAGTTAGAAG
GAATGATCTT.AATCTTACATTGGAGCCAAATCGCCAATACACTCATGCCACATGGGATG
CTTCCTTTTAAATTATTAA

> DcaMADS28

ATGGGGAGAGGAAAAATAGAGATAAAAAGAATAGAAAACTCAACAAATAGGCAAGTT

ACATATTCAAAAAGAAGAAGTGGAATTATTAAAAAAGCTACAGAAATTACAGTTCTTTG
TGATGCTAAGGTTTCTCTTATTATTTTCTCTAATAATGGAAAAATGCATGCTTATCATAGT
GCTGGATCTTCGGTTGAAGAAATCCTAGATCAATACCACAAGATCTCTGGGAAAAGAC
TTTGGGATGCAAAACATGAAAATCTCAGCCATGAGATTGACCGAGTCAAGAAAGAAA
ATGAGAACATGCAGATTGAGCTGAGGCACTTGAAGGGAGAGGACATCCAATGTTTGCC
ATATCCAGATTTGATGAGGCTTGAAGATGCTCTTGAAAATGGTCTTATCGGTATCCGTGA
AAAACAGATGGAGATCTACAAGATGCACAAAAAAAATCATAGGATGCTTGAGGAGGA
GAATAATCAGCTTGTATACATGTTGCACAAGCAAGCAGAGATGGAGGCAGGTGTATGC
AGCAACAACAACAACTACGATCAGCACCCGATTCCATCGTTCGGGTTTCGGGTTCAAC
CCATGCAACCCAACCTCCAAGATCGGCTCTAA

> DcaMADS29

ATGGCAAGGGGTAAAATACAGATAAAAAAAAATAGAAAATCTAACAAATAGGCAGGTGA
CATATTCAAAGAGAAGAAATGGATTGTTTAAGAAGGCAAATGAATTAACTGTGTTGTGT
GATTCTACTGTTTCTATTATTATGTTGTCTAGTAATAATAAGTTGCATGAGTTTCTTAGCC
CTGCTTCTAATCTCACGACCAAGGACGTTTATGATAGGTATCAGAAGGTGTTAGGAATT
GATATATGGGTCACTCATGAGAAGAAAATGCAAGAACAATTGAGGAAGTTAAATGAGG
ATAAGAGGAACCTTCAAATAGAAATCAGGCGAAGAATGGGGGACTGTTTGGAGGATTT
GAGCTTCCATGAACTGTGCATTCTTGGGAATGAAATGGAGAATGCTTCGACCCTCATTC
GAGAACGCAAGTACAAGAAGATCGATGGTCAGATCGACACTACAAAGAAAAAGGTGC
GAAATGGGAATGAAATTCACAAGGGTCTCCTTCAAGAATTTGAAATGCCGAGAGAAG
AACCGCAGTATGGACTAGTTGACAACGGAGAGTACAACAGTATGCTCAGCTACAACGA
CGCTCGTCTGCTCGCTCTCAGATTGCAGCCATGTCAGCCTAACATTCACACCGGTGCAG
GCTCAGGATCGTGTGTCACGACTTACACACTGCTCTAA

Figure S1. The ORF of DcaMADS6, DcaMADS8, DcaMADS18, DcaMADS28, and DcaMADS29.

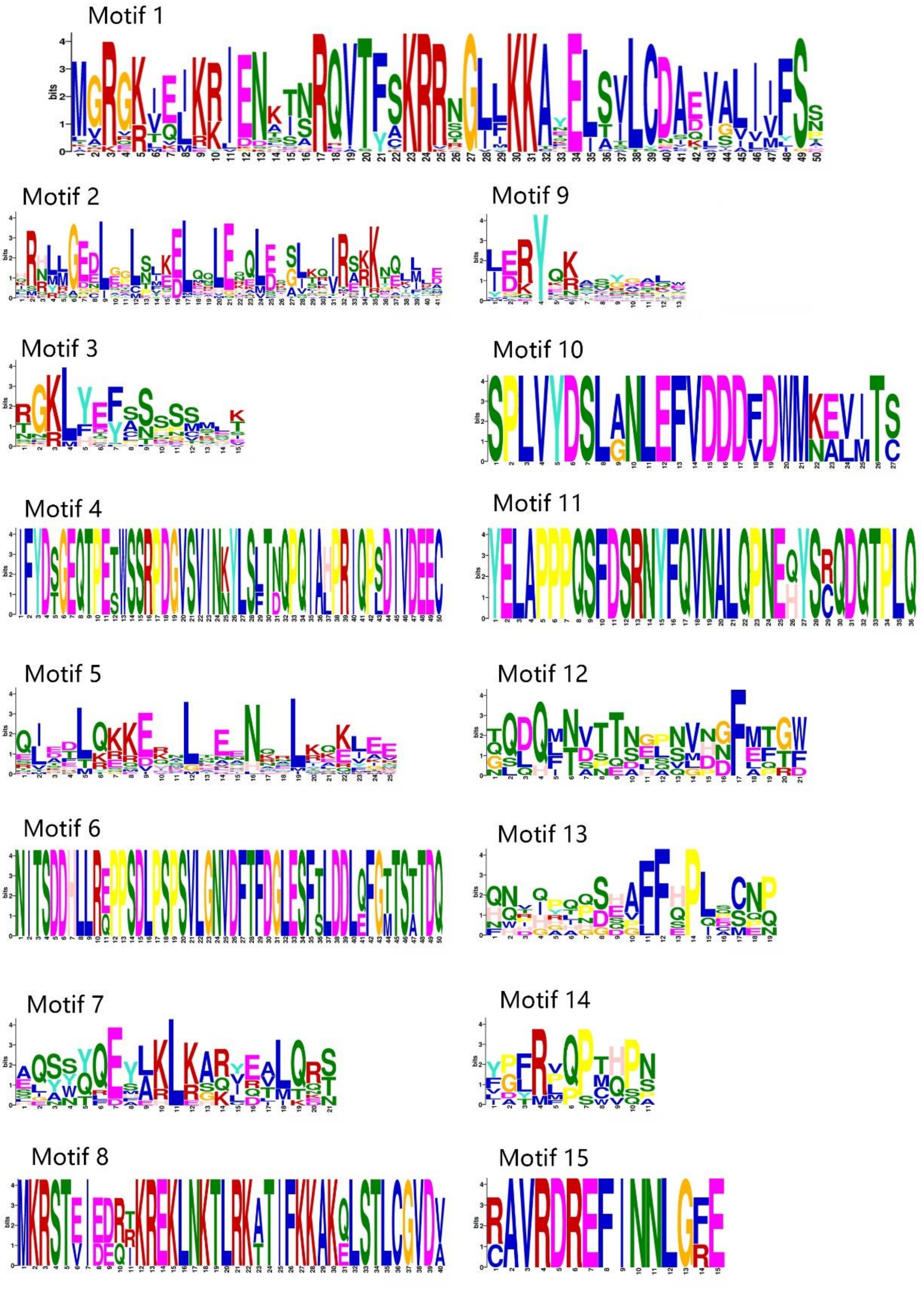

Figure S2. Conserved motifs of 39 carnation MADS-box proteins identified using the MEME program. The information of Motifs 1 to 15.

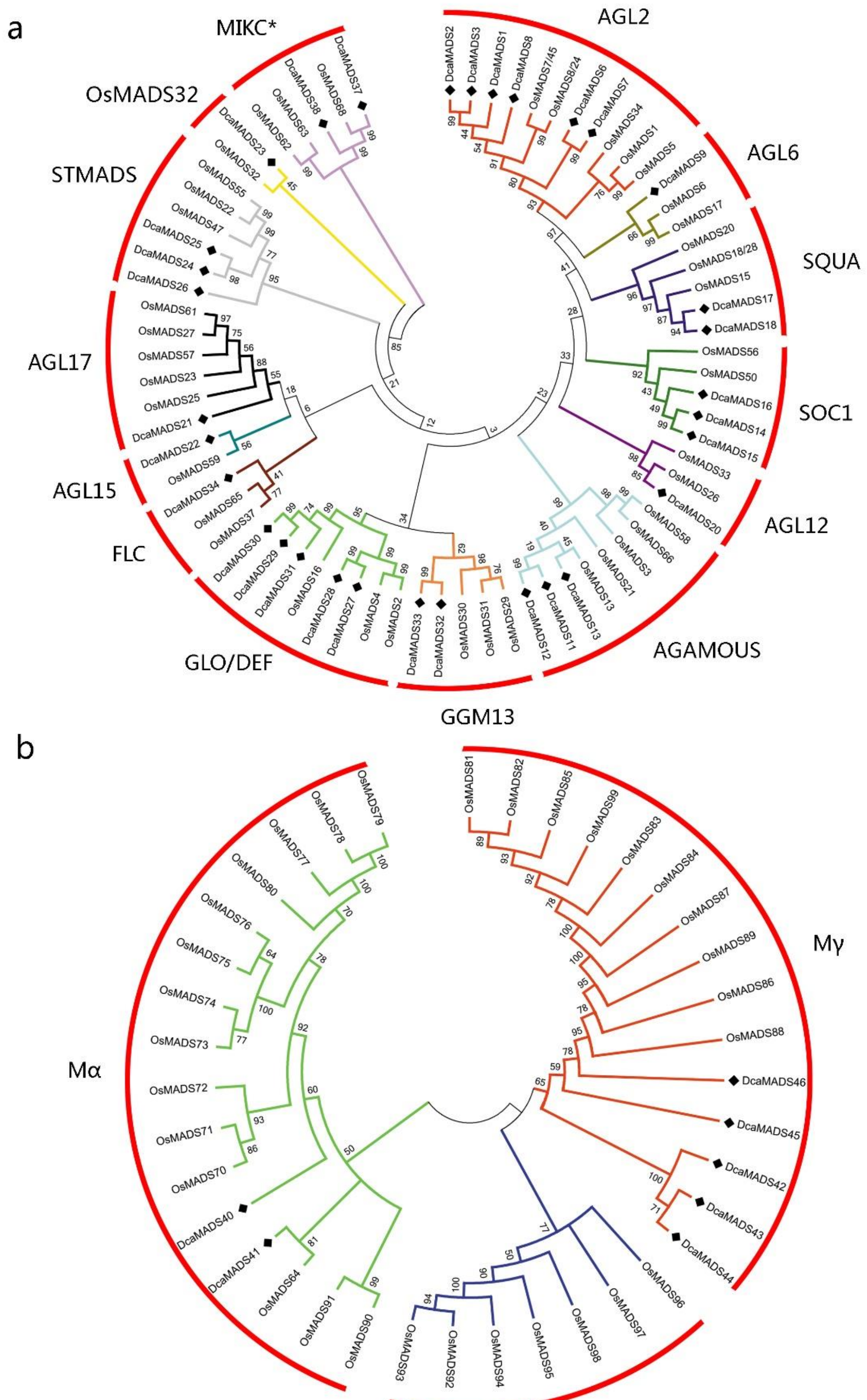

Figure S3. The phylogenetic tree of the 39 DcaMADS genes was generated by the neighbour-joining (NJ) algorithm using Molecular Evolutionary Genetics Analysis (MEGA version 6.0) software. The subgroups are marked in different colors. (**a**) Phylogenetic tree of *D. caryophyllus* and rice type II proteins. (**b**) Phylogenetic tree of rice and *D. caryophyllus* type I proteins.

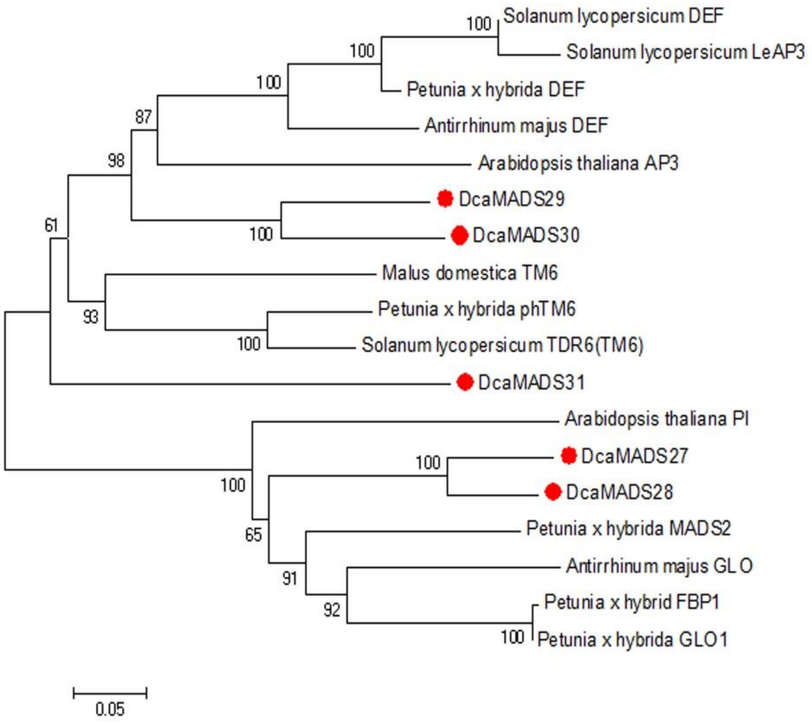

Figure S4. The phylogenetic tree of the class B genes in carnation was generated by the neighbour-joining (NJ) algorithm using MEGA (version 6.0) software.

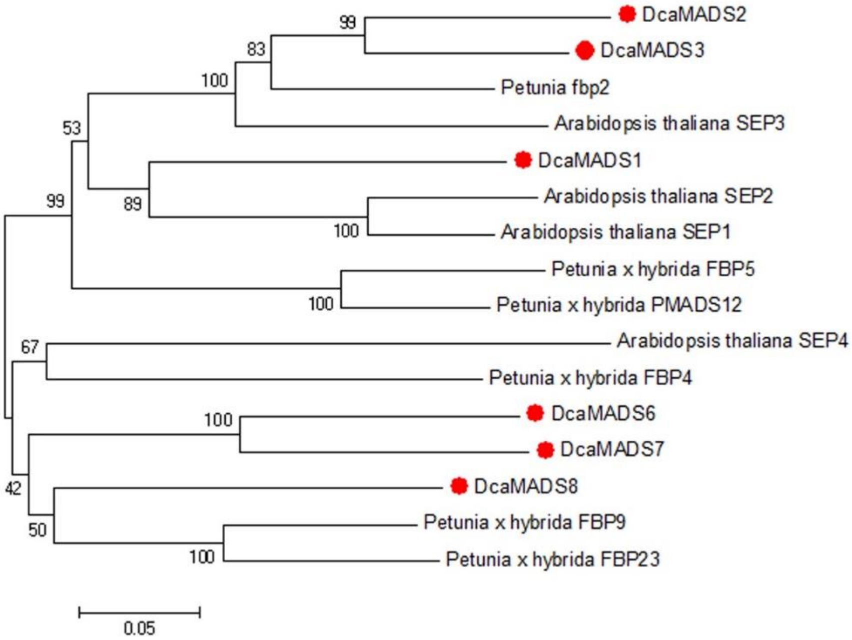

Figure S5. The phylogenetic tree of the class E genes in carnation was generated by the neighbour-joining (NJ) algorithm using MEGA (version 6.0) software.

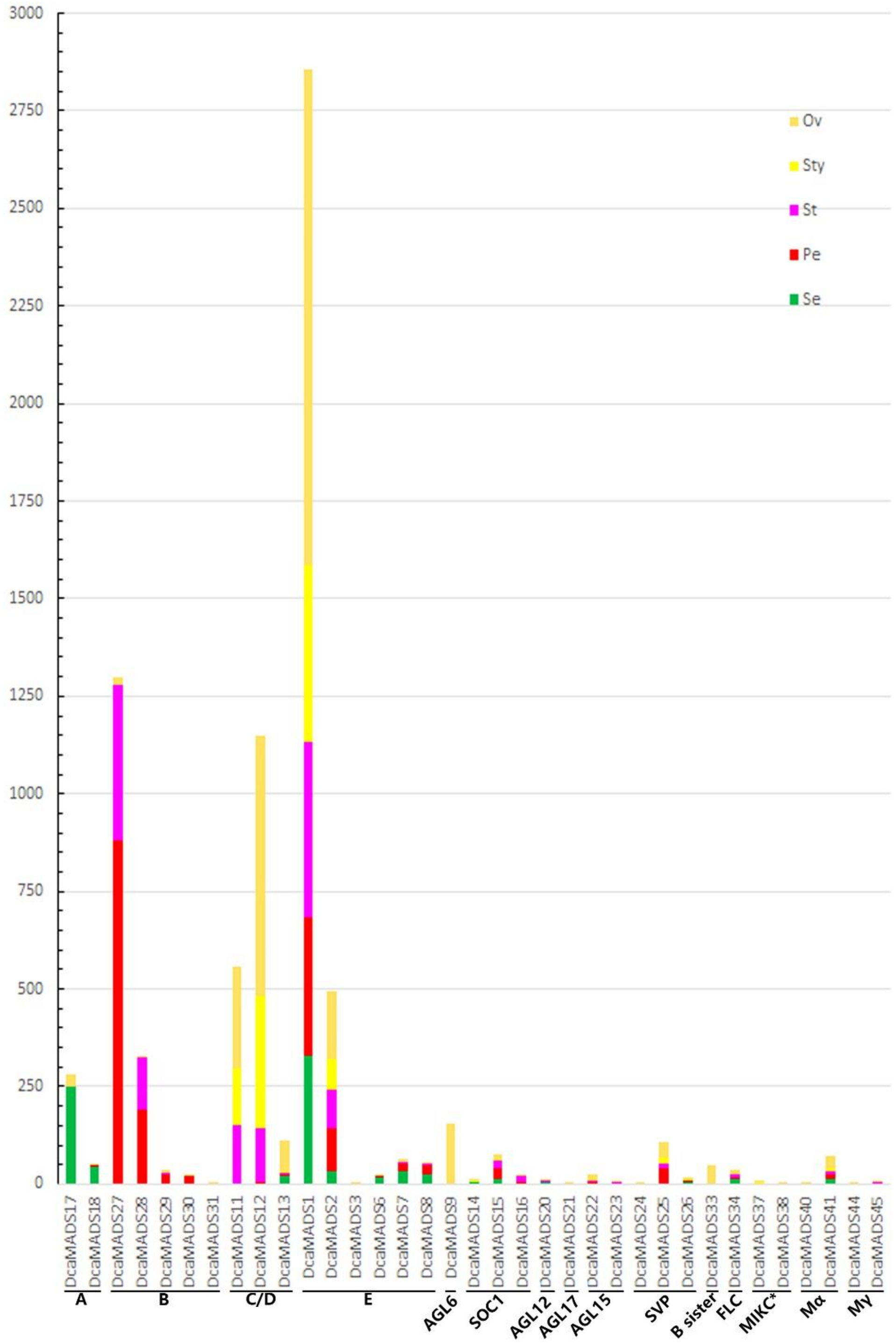

Figure S6. Organ specific expression of 35 DcaMADS genes at different flower whorls. Se: sepals, Pe: petals, St: stamens, Sty: styles, Ov: ovaries.
